# Comparison of individual and ensemble machine learning models for prediction of sulphate levels in untreated and treated Acid Mine Drainage

**DOI:** 10.1007/s10661-024-12467-8

**Published:** 2024-03-02

**Authors:** Taskeen Hasrod, Yannick B. Nuapia, Hlanganani Tutu

**Affiliations:** 1 Molecular Sciences Institute, School of Chemistry, University of the Witwatersrand, Private Bag X3, Johannesburg, 2050 South Africa; 2 Pharmacy Department, School of Healthcare Sciences, University of Limpopo, Turfloop Campus, Polokwane, 0727 South Africa

**Keywords:** Acid Mine Drainage, Sulphate, Machine learning, Regression, Stacking ensemble machine learning, Environmental chemistry

## Abstract

Machine learning was used to provide data for further evaluation of potential extraction of octathiocane (S_8_), a commercially useful by-product, from Acid Mine Drainage (AMD) by predicting sulphate levels in an AMD water quality dataset. Individual ML regressor models, namely: Linear Regression (LR), Least Absolute Shrinkage and Selection Operator (LASSO), Ridge (RD), Elastic Net (EN), K-Nearest Neighbours (KNN), Support Vector Regression (SVR), Decision Tree (DT), Extreme Gradient Boosting (XGBoost), Random Forest (RF), Multi-Layer Perceptron Artificial Neural Network (MLP) and Stacking Ensemble (SE-ML) combinations of these models were successfully used to predict sulphate levels. A SE-ML regressor trained on untreated AMD which stacked seven of the best-performing individual models and fed them to a LR meta-learner model was found to be the best-performing model with a Mean Squared Error (MSE) of 0.000011, Mean Absolute Error (MAE) of 0.002617 and *R*^2^ of 0.9997. Temperature (°C), Total Dissolved Solids (mg/L) and, importantly, iron (mg/L) were highly correlated to sulphate (mg/L) with iron showing a strong positive linear correlation that indicated dissolved products from pyrite oxidation. Ensemble learning (bagging, boosting and stacking) outperformed individual methods due to their combined predictive accuracies. Surprisingly, when comparing SE-ML that combined all models with SE-ML that combined only the best-performing models, there was only a slight difference in model accuracies which indicated that including bad-performing models in the stack had no adverse effect on its predictive performance.

**Supplementary Information:**

The online version contains supplementary material available at 10.1007/s10661-024-12467-8.

## Introduction

Water is a basic necessity that every living creature depends on (Westall & Brack, 2018). Access to clean water for drinking and sanitation purposes is a basic human right (Gleick, 1998) and its importance is further emphasised in its own dedicated United Nations Sustainable Development Goal (SDG) number 6 which works in tandem with all other SDGs since none of them can be achieved without the access to safe and adequate water (Mirumachi & Hurlbert, 2022).

However, in order to meet current and future water demands, sustainable wastewater treatment processes must be implemented to treat wastewater and reclaim clean water as well as valuable by-products (Smol et al., 2020). In countries like South Africa, wastewater such as Acid Mine Drainage (AMD) produced from mining related activities poses a huge threat to human, wildlife and environmental health due to its toxicity and heavy metal content (McCarthy, 2011; Naidu et al., 2019). Several processes are in place to reduce the toxicity of AMD such that it can be discharged into nearby rivers and streams (Dhir, 2018; Johnson & Hallberg, 2005a). However, high sulphate concentrations persist in treated AMD and on discharge, can undergo a series of complex redox reactions that could result in the reduction of sulphate to elemental sulphur or octathiocane (S_8_) which precipitates out in basic reducing environments such as wetlands (Kushkevych et al., 2021; Steudel, 1996). This octathiocane is the pharmaceutically active form of sulphur which is used in many cosmetic applications as an antiseptic and disinfectant (Carretero & Pozo, 2010). It is, therefore, an economically viable by-product to extract from both treated and untreated AMD.

Traditional analytical chemistry approaches used to measure sulphate levels in AMD such as turbidity, ion-chromatography, inductively coupled plasma atomic emission spectroscopy (ICP-AES), spectrophotometry, gravimetry and titrimetry are time-consuming, expensive, utilize specialized equipment and make use of hazardous chemicals (Reisman et al., 2007; Roy et al., 2011). Therefore, sulphate concentrations may be inadequately determined, resulting in missing values in large datasets.

In order to design experiments to extract octathiocane from AMD, large amounts of accurate AMD sulphate concentrations are needed for the design and simulation of experiments using customized software such as PHREEQC or Geochemist’s Workbench. However, the challenge is that such sulphate concentrations are at present missing from large water quality datasets due to the issues highlighted above and as such, no practical simulations can be run to determine the optimal conditions for reducing sulphate to precipitate out the economically valuable octathiocane.

In order to overcome the challenge outlined in the previous paragraph, we have employed machine learning techniques to predict the sulphate levels in AMD. Machine learning (ML) provides a suitable approach to address these challenges due to its incredibly fast predictive time, high accuracy and cost-effective nature since it can be used as an alternative to conducting the sulphate-determining experiment and, therefore, removes the hazards, costs and time associated with the experiment. This was done by using the ability of ML models to learn the patterns present in water quality datasets and understand the relationships between the desired sulphate levels and its corresponding water quality parameters in order to predict the sulphate levels.

ML is a sub-field of artificial intelligence (AI) that uses statistics and mathematics to learn patterns in large datasets over time, to identify hidden patterns (Zhong et al., 2021) and to deliver key insights (Alzubi et al., 2018). ML has gained significant popularity over the last few years due to its ability to rapidly deliver accurate results. When looking at environmental assessment, ML has successfully been used, for example, to predict ammonia levels in groundwater to understand the nitrogen reduction pathways and nitrogen conservation and loss and found that four main factors explained 70.97% of the variance (Perović et al., 2021). An adaptive neuro-fuzzy inference system was used to predict the reaeration coefficient of streams in India and found that there were multiple sources of organic loading in the urbanized river, which would allow for waste and water resource management schemes to be developed (Arora & Keshari, 2023). ML was used to model soil moisture and its effect on slope stability in order to identify triggers of shallow slope landslides and found that for a real event, the triggering time was correctly predicted using the developed model (Bordoni et al., 2018). One study used a forward-feeding dynamic neural network to develop an early warning system for reservoir water management and maintenance and was able to forecast the total phosphorous and total nitrogen content of the waterbody (Wang et al., 2021). One study looked at comparing the performance of a single decision tree with a bagged decision tree ensemble and a least-squares boosted tree and found that the bagged and boosted trees performed the single tree when modelling the organic matter present in water (Tahraoui et al., 2022). In another study, it was shown that an electrochemical system when used in conjunction with ML was more efficient at removing antibiotics from wastewater than traditional electrochemical systems (Foroughi et al., 2020). A double machine learning model and time series clustering were shown to be effective in assessing and understanding climate change and lake response in China, and this study showed that precipitation was the most important feature for lake area growth rate (He et al., 2023). In another study aimed at assessing the determinants of environmental sustainability, ML was used to determine the factors associated with CO_2_ emissions and their related correlations (Jabeur et al., 2022). The findings showed that advanced and interpretable machine learning models were successfully used to predict CO_2_ emissions from large panel data which assists organizations and policymakers in understanding data such that they can develop energy reduction policies and improve environmental quality (Jabeur et al., 2022). The versatility of ML has seen it outperform some traditional modelling approaches, for instance in the prediction of groundwater levels. This has proven to be cost-effective for sustainable crop production since the findings showed that the proposed modelling approach can provide important information about groundwater variability at a short time scale (Guzman et al., 2019).

When looking at sulphate levels particularly, it was found that an artificial neural network could easily predict sulphate levels in drinking water with a high degree of accuracy due to the nonlinear physio-chemical relationships that existed between sulphate and the other water quality parameters (Tahraoui et al., 2021). In another study, Gaussian Process Regression (GPR) was successfully used to accurately predict sulphate levels in raw water and was found to be suitable to be used as a continuous water monitoring model due to its ability to deal with outliers, missing values and update itself (Tahraoui et al., 2022). The GPR model was then used to efficiently predict the dissolved organic carbon, ultraviolent absorbance at 254 nm and turbidity reduction rates (Tahraoui et al., 2023).

When focussing specifically on AMD assessment, ML techniques have gathered much attention due to their robustness and generalizability in various countries with there being a strong focus on combining earth observation data with machine learning to promote sustainable development (Ferreira et al., 2020). ML image recognition techniques, for instance, were applied to monitor mining environmental problems using aerial drone technology in Ziyang County, China using SVR, RF and U-Net Neural Network and found that mine environmental problems could be detected by combining ML models with drone technology (Kou et al., 2023). ML techniques such as Bayesian machine learning and functional data analysis (FDA) were used in Fabero, Spain in order to detect anomalous river flow activities that would serve as early flood warning signs and found that the created machine learning tool can identify anomalies and automatically capture the pH patterns (Rigueira et al., 2023). In northern Tunisia RF, SVM and MLP were used to predict toxic heavy metal content and the behaviour of mine waste in relation to the dissolution of iron-bearing minerals and found that heavy metal concentrations and sulphate dissolution increase as the oxidation of sulphate-bearing minerals such as pyrite increases (Trifi et al., 2022). RF, XG and ANN were used to create a web application that predicted electrical conductivity and pH in Johannesburg, South Africa in order to improve forecasting procedures that ensure the smooth operation of AMD treatment plants (More & Wolkersdorfer, 2022). In one study in the Philippines, a neural network with particle swarm optimization was used to improve mapping prediction capabilities for spatial maps to predict ground water quality (De Jesus et al., 2021). The extent of AMD pollution was mapped using aerial drone technology, RF and hyperspectral technology in the Tintillo River, Spain in order to better evaluate contaminated water bodies and the results showed that dissolved metals could be predicted and iron species could be traced within sediments (Flores et al., 2021). The results were also found to be more comprehensive than those based on traditional remote sensing only due to faster data acquisition times and non-invasive methods (Flores et al., 2021). In another study, a neural network was used to predict mine seepage flow rate in Canada to improve flood control, mine site management and contaminant treatment strategies that rely on weather and rock dynamics as well as climate change and found that ANNs were able to investigate long-term weather trends (Ma et al., 2020). Neural networks and SVM using a probability bound analysis have also been used to identify and quantify uncertainties related to AMD in order to mitigate risks and improve AMD management strategies and the findings showed that ANNs outperformed SVMs (Betrie et al., 2014). In another study, a neural network, SVMs, DT and KNN were used to predict copper concentrations in an AMD plant to develop cost-effective and sustainable AMD remediation strategies and found that the best model for predicting copper concentrations was a SVM with a polynomial kernel (Betrie et al., 2013).

After a thorough literature review and to the best of our knowledge at present, it was found that there were no publications regarding the use of machine learning to predict sulphate values in particular in AMD. Additionally, there were also no publications on the use of stacking ensemble machine learning regression algorithms for water quality prediction in AMD treatment plants. Also noted, was the lack of versatility in models used to predict AMD water quality parameters since the models mainly used were neural networks, SVM and RF. A novel approach to predicting water quality in AMD would be to use a range of diverse ML regression algorithms and also to combine heterogenous (i.e. different) regression models using a stacking ensemble regressor (SE-ML). Additional novelty to this would be to explore different architectures pertaining to how heterogenous models are stacked and its effect on the overall predictive performance of the model. Additionally, the use of computational methods (ML to provide data for geochemical modelling) to design reduction experiments for the extraction of octathiocane from AMD is novel.

Therefore, this study explored the use of individual ML regression models and then stacked these in an ensemble architecture to overcome the challenge of inadequacies that individual models are usually prone to. By stacking these models, the robustness and prediction capability of the whole usually supersedes that of the individuals, a phenomenon usually referred to as “the wisdom of the crowds”. The developed and accurately trained model can now be deployed to different AMD treatment plants to predict the necessary sulphate levels as a pre-cursor for further remediation studies to assess the feasibility of recovering octathiocane from AMD.

## Methodology

### Hardware and software used

The software used for all data preparation processes and machine learning was Python 3.11 programming language hosted in the Google Colaboratory environment using a Lenovo V110 laptop equipped with Intel® CoreTM i3-6006U CPU @ 2.00 GHz. Geochemical modelling was conducted as an auxiliary data-cleaning step using PHREEQC Interactive 3.7.3–15,968 (Parkhurst & Appelo, 2013).

The specific Python packages (with their version numbers in brackets) used are detailed as follows: Numpy (1.23.5) and Pandas (1.5.3) were used for data cleaning, processing, conversion and exploratory data analysis. Scikit-learn (1.2.2) was used for model training and testing. Graphing and visualizations were conducted in Matplotlib (3.7.1), Seaborn (0.12.2) and Plotly. (5.15.0).

### Location and acquisition of data

Historical data collected over the years of 2015–2020 at the Central Rand AMD treatment plant in Johannesburg, South Africa was obtained. This dataset consisted of water quality parameters measured at three locations in the plant, “Pump A” and “Pump B” which were untreated AMD and “Treated Water” which was sampled at a location after the treatment process. The water quality parameters measured for each location were Temperature (°C), Electrical Conductivity (mS/m), Total Dissolved Solids (mg/L), pH, Turbidity (NTU), Total Suspended Solids (TSS) (mg/L), Sulphates (mg/L), Aluminium (mg/L), Iron (mg/L) and Manganese (mg/L). The historical data covers continuous monitoring over the years 2015–2020 with daily analysis being conducted on three samples, i.e. one from each of the three locations.

### Data processing

The initial large Central Rand dataset was firstly broken down into three smaller datasets according to the sampling site and these datasets were each dealt with individually for all the following procedures and ML. These datasets will hereafter be referred to as “A-” (Pump A), “B-” (Pump B) and “T” (Treated Water) for convenience. The aluminium, TSS and turbidity columns were missing in most instances and were, therefore, dropped from all datasets. The date column was also dropped since it provided no meaningful information pertaining to the water quality. Individual water quality parameter headings were shortened to “TEMP”, “EC”, “TDS”, “pH”, “SUL”, “FE” and “MN” with the prefix “A- “, “B- “ or “T- “ indicating each of the three categories. The units were also removed for each dataset due to cluttering, however, the units for each water quality parameter remain the same as indicated under “Location and Acquisition of data”. Any values that had a “ ± 5%” associated with them were fixed by removing the “ ± 5%” using Excel’s built-in “Find and Replace” feature.

The smaller datasets were then subjected to data pre-cleaning where blank rows and character data signifying breakdowns or plant shutdowns were removed. For all parameters, numerical typos such as recording “6..19” instead of “6.19” were fixed, “Not-a-Number” (NaN) values were removed and all remaining data was converted to a float.

Geochemical modelling was then conducted on the pre-cleaned datasets as an additional cleaning step in order to thermodynamically correct for any inaccuracies by performing a charge balance on the sulphate levels using the TEMP, pH, Fe and Mn values. Several outliers and incorrect data points were identified here and were further removed from the datasets in order to minimize any noise present since water quality is inherently stochastic. Specific outliers removed were those on which the total anionic species concentration did not balance with the cationic species concentration or where the pH recorded would be unreasonable when looking at the measured concentrations of ionic species present.

The datasets then underwent data cleaning and Exploratory Data Analysis (EDA) in Python. Data cleaning consisted of removing any outliers as identified using the Inter Quartile Range method (to further minimize any noise) and normalizing the data (using Python’s “MinMaxScaler”) in order to reduce the effects of the differing scales (i.e. units). EDA used statistical distributions of the data and visualizations to understand the relationship between variables. Visualizations used were histograms, density plots, box and whisker diagrams, scatter matrices and correlation matrices before and after outlier removal. The units for each of the water quality parameters were omitted from the graphs in order to prevent cluttering. From the box and whisker diagrams, it was found that many outliers existed that were well above and below the minimum and maximum values, respectively. As such, the Inter Quartile Range method was deemed to be safe in determining which data points constitute the main dataset and which datapoints constitute outliers since anything above 1.5 times the upper quartile and 1.5 times the lower quartile falls outside the main dataset and becomes an outlier. It is necessary to remove outliers since they heavily skew the distribution of the data and, therefore, contaminate the statistical representation of the datasets which would lead to poor model performance when conducting machine learning.

Feature extraction using Principal Component Analysis (PCA) and Recursive Feature Elimination (RFE) was then conducted to reduce the dimensionality (i.e. reduce the number of predictors) in the data and limit overfitting. PCA was conducted using two principal components and its results were visualized using a bi-plot in order to determine the inter-relationships between variables by observing clustering of individual observations (scores) and how the water quality parameters were related to each other (i.e. loadings). The number of optimal clusters was determined using the “Elbow Method” which deems the optimal number of clusters to be the point at which a plot of “Number of Clusters” against the “Within-Cluster Sum of Squares” bends and a “K-Means” clustering model was used to determine the actual clusters. A Scree plot was also used to determine the number of principal components that were necessary to explain at least 75% of the variance in the data. RFE was conducted using LR as the fitting model and the “number of features selected” was the number of principal components needed to explain at least 75% of the variance (which was found using the Scree plot). The highest ranking features, as determined from RFE, were then deemed to be the most important features and were selected to be the predictors and combined with the sulphate values (target) in order to create the final datasets ready for ML.

### Machine Learning

Each of the A, B and T datasets were individually dealt with for ML by splitting them into an 80%:20% Train:Test split. In addition, 10-fold cross-validation was used to assess the performance of the model in a more reliable and robust manner. The regression models used in this study along with their mathematical descriptions and explanations can be found in Table 1. The experimental settings (hyperparameters) used for each of the models mentioned in Table 1 were the default settings of the models when imported from Python’s Scikit-learn library. No modifications to these hyperparameters were made. For the random forest (RF) regressor, the number of estimators used was 100 in all instances. This was done on purpose in order to fairly compare the predictive performance of all the models. Optimisation of the models was deemed to be unnecessary since the default settings provided accurate predictions. As a rule of thumb, though, this optimisation (or hyperparameter tuning) for RF ranges from 50 to 400 trees. Generally, adding trees reduces overfitting, owing to the bagging and random feature selection capability of RF.

**Table 1:** Machine learning regression algorithms used to predict sulphate levels and their mathematical explanations

Regression model	Explanation and equation	References
Linear (LR)	This model is used when the relationship between the predictor and the target is linear. It can be further broken down into two main types based on the number of predictors, namely, simple linear regression where there is only one predictor and multiple regression analysis where there are multiple predictors This model simply uses the equation of a straight line in the form, $y= {\beta }_{0}+ {\beta }_{1}x+ \varepsilon$ where *y* is the target, *x* is the predictor, ${\beta }_{1}$ is the gradient, ${\beta }_{0}$ is the y-intercept or bias and $\varepsilon$ is the error. The line of best fit is determined by varying m and c in order to minimize the error between the observed and predicted values. This model is sensitive to outliers in the dataset and, therefore, should not be used on large data sets.	Maulud and Abdulazeez (2020)
LASSO	“LASSO” stands for “**L**east **A**bsolute **S**hrinkage and **S**election **O**perator” and shrinks data values towards a central point, e.g. the mean. It is also known as L1 and performs feature extraction along with parameter estimation. This is due to the shrinkage of some feature coefficients to zero using the tuning parameter λ, which allows for feature extraction from the dataset. ${\widehat{\beta }}_{{\text{LASSO}}}={\text{argmin}} \left\{\frac{1}{2}\sum_{i=1}^{N}({y}_{i}-{\beta }_{0}- \sum_{j=1}^{p}{x}_{ij}{\beta }_{j}{)}^{2}+ \lambda \sum_{j=1}^{p}\left|{\beta }_{j}\right|\right\}$ It is worth noting that this is the same as minimizing the sum of squares of errors with a tuning parameter (λ) to control the shrinkage. Over-fitting is reduced since only required features are used and other features are set as zero. For highly collinear variables, only one variable is picked and the rest are shrunk to zero. This model works well for data that has few variables.	Muthukrishnan and Rohini (2016)
Ridge (RD)	This is also known as L2. When there is high bias in the data set due to multicollinearity this technique is used and is also known as Regularization. The λ (lambda) in the equation below reduces the multicollinearity: ${\widehat{\beta }}_{{\text{ridge}}}={\text{argmin}} \left\{\sum_{i=1}^{N}({y}_{i}-{\beta }_{0}- \sum_{j=1}^{p}{x}_{ij}{\beta }_{j}{)}^{2}+ \lambda \sum_{j=1}^{p}{{\beta }_{j}}^{2}\right\}$ Whilst both LASSO and Ridge regression constrain coefficients using a penalty factor, LASSO takes the magnitude of the coefficients whilst Ridge takes the square of the coefficients. Ridge should be used when there is high correlation between variables when bias and over-fitting are high. Ridge is not suitable when the number of predictors is too large.	Muthukrishnan and Rohini (2016)
Elastic Net (EN)	This model performs regularization and feature selection simultaneously by combining the penalties from LASSO and Ridge and learning from their shortcomings to improve its regularization. It improves on LASSO’s limitation of only picking one feature when there is multicollinearity in the dataset. Then the EN penalty is, ${\widehat{\beta }}_{EN}= \frac{1}{2n}\sum_{i=1}^{n}{\left({y}_{i}-{\beta }_{0}- \sum_{j=1}^{p}{x}_{ij}{\beta }_{j} \right)}^{2}+\lambda \left(\frac{1-\alpha }{2}\sum_{j=1}^{p}{{\beta }_{j}}^{2}+\alpha \sum_{j=1}^{p}\left|{\beta }_{j}\right|\right)$ where α is a mixing between 0 and 1 used to adjust the weighting of the contribution of each penalty (Ridge = 0 and LASSO = 1) to the loss function. This is a powerful technique to be used when the number of predictors (*p*) is greater than the number of samples used. The double shrinkage of coefficients can cause low efficiency in predictability and high bias.	García-Nieto et al. (2021)
k-Nearest Neighbours Regression (KNNR)	This algorithm works by picking a certain point and evaluating its “k” nearest neighbours to find similarities and, thereby, grouping points together based on their similarities. The algorithm works on the bases of evaluating distances between points with the assumption that similar data points lie closely to each other. Thus, the distances between points that are deemed to be “similar” are minimized and the distance between cluster that are deemed to the “different” are maximized. By training a regression function (*f*), targets (*y*_*i*_) are found using a set of predictors (*x′*) from a set of *N* observations where N_k_(x*′*) contains the indices of the k nearest neighbours of x*′.* ${f}_{KNN}\left({x}{\prime}\right)= \frac{1}{k} \sum_{i \epsilon {N}_{k({x}{\prime})}}{y}_{i}$ This algorithm does not perform well on large datasets since it is heavily reliant on memory to store all of its data during training. It also does not perform well on high dimensionality data and can be prone to overfitting.	Kramer (2013)
Support Vector Regression **(SVR)**	A hyperplane (i.e. the straight line required to fit the data) is constructed such that data lies on either side of it. The data points that lie closest to the hyperplane are known as the support vectors and these influence the position and orientation of the hyperplane. This hyperplane is used to predict discrete values and find the line of best fit using a threshold value instead of minimizing the error between the real and predicted value. This threshold value is the distance between the hyperplane and the support vectors (*w*) SVRs are governed via the mathematical equations shown below which constitute the minimization problem: $$f\left(x\right)= {\left[\begin{array}{c}w\\ b\end{array}\right]}^{T}\left[\begin{array}{c}x\\ 1\end{array}\right]= {w}^{T}x+b x,w\epsilon {\mathbb{R}}^{M+1}$$ ${{\text{min}}}_{w}\frac{1}{2}{\Vert w\Vert }^{2}$ It is also tolerant towards outliers and can easily be updated. However, these models are not suitable for larger datasets and for datasets that contain a lot of noise.	Awad and Khanna (2015)
Decision Tree Regressor (DT)	This is a non-linear regressor model that consists of a hierarchy of nodes that are easy to interpret. At each node, a mathematical decision is evaluated to “True” or “False” simply by determining whether a sample follows this rule. This is similar to an if-else ladder and leads to a sample transversing the depth of the tree via a series of True–False decisions taken. The Tree starts at the root rode whereby only a single decision is taken. Thereafter, the sample is passed through a series of decision nodes and finally terminates at the leaf node where a final decision and, hence, a final value ($\overline{{Y }_{t}}$) is given as the output. If *Y*_*i*_ is the initial split value and *n* is the number of a value at the current node, $\overline{{Y }_{t}}=\frac{1}{n}\sum_{i\epsilon n}{Y}_{i}$ This model is extremely susceptible to small variations the training data and is prone to overfitting.	Pekel (2020)
Extreme Gradient Boosting Regressor (XG)	This is a collection of *sequential* decision trees that are trained upon each previous tree’s mistakes in order to try and correct them. The final learner is essentially a collection of each of the previous “weak” learners which were refined and able to form a “strong” learner, whereby, a final accurate prediction is given. XG also suppresses weights by applying advanced regularization techniques. The loss function calculated to minimize the error between the true and predicted values is given as follows: ${L}^{(t)}= \sum_{i=1}^{n}l\left({y}_{i} , {\widehat{y}}_{i}^{(t-1)}+{f}_{t}({x}_{i})\right)+\Omega ({f}_{t})$ where *l* is the loss function, *t* is the number of iterations and *Ω* is the decision tree complexity penalty. This algorithm reduces the overfitting present in decision trees and works well when there are imbalanced classes. However, it is a complex algorithm that requires a significant amount of hyperparameter tuning.	Kavzoglu and Teke (2022)
Random Forest Regressor (RF)	This is essentially a collection of *parallel* decision trees that utilizes bagging and bootstrapping. Bagging is simply an ensemble learning approach whereby several decision trees are collected and trained in parallel at the same time using different sub-sample of the data in order to reduce overfitting. Bootstrapping randomly samples subsets over a given number of iterations and averages the results to obtain an even better result. Random forest regression is governed by the mathematical equation, $Y= {E}_{0}(X,{\theta }_{k})$ where *k* indicates the number of trees planted depending on the random variable (*θ*), *X* is the given input, *Y* is the desired output which is calculated from taking the average over all the trees. This model works well with non-linear data; however, the model is prone to over-fitting and is not easily interpretable.	Liu et al. (2012)
Multilayer Perceptron Artificial Neural Network (MLP)	These networks are inspired by the neurons in the brain and mimic the way neurons signal to each other. They are comprised of mode layers where input nodes are connected to output nodes via a series of hidden layers. Nodes are connected to each other via a series of weights and thresholds. Node activation is responsible for sending data to the next layer of the network and occurs when the output of the individual node is above the threshold value. They make use of the following mathematical equation to minimize an error via the updating of weights. $\widehat{y}=sign\left\{\overline{W}\bullet \overline{X}\right\}=sign\left\{\sum_{j=1}^{d}{w}_{j}{x}_{j}\right\}$ where $\overline{X }$ is the sum of input features, *d* is the number of nodes, $\overline{W }$ is the sum of weights, $\widehat{y}$ is the predicted value and $\overline{W }\cdot \overline{X }$ is the linear function which is computed at the output node. Node activation occurs when the *sign* is positive. These models are able to store large volumes of data and work with incomplete data. They also have a high fault tolerance and parallel processing capabilities. However, these models require high computing power due to their parallel processing and are sometimes hard to explain since their behaviour is sometimes unknown.	Vivanco-Benavides et al. (2022)
Stacking-Ensemble Machine Learning (SE-ML)	Stacking ensemble machine learning (SE-ML) involves the combination of predictions from multiple heterogeneous machine learning models on the same dataset. A single model learns how to best combine the predictions from each of the individual models, thereby, using the “wisdom of the group”. In this way, one hopes to remove the errors associated with each individual model and obtain a much more accurate representation of the true value. SE-ML trains heterogenous (i.e. different) base models with a training set in the first layer (level 0) to obtain the crucial features from the dataset. Thereafter, these crucial features and individual predictions are combined and fed as the input features to train a meta-learner (level 1) model in the next layer in order to give the final prediction. For regression problems, the level 1 regressor is LR.	Hu et al., (2020a, 2020b), Mallick et al. (2022), Hu et al., (2020a, 2020b)

For training, all regression models mentioned in Table 1 were used to create a baseline for comparison and evaluated using the Negative Mean Squared Error (NMSE) metric. To determine the effect of combining heterogenous models, SE-ML was firstly conducted by combining all the regression models, thereafter, it was conducted using only the best-performing models (as determined from the baseline) to determine the effect of removing poorly performing models from level 0 in the stack. LR was used as the level 1 meta-model in both cases. SE-ML was also evaluated using NMSE during training.

For testing, all baseline models and both stacks were compared to each other and evaluated using the Mean Squared Error (MSE), Mean Absolute Error (MAE) and Coefficient of Determination (*R*^2^) metrics in order to determine the best-performing model for each dataset A, B and T.

## Results and discussion

### Pump A

The sulphate values initially lied in the range of 3000–4000 mg/L, however after geochemical modelling the sulphate values dramatically decreased and now lied in the range of 0.002–0.0015 mg/L which was five orders of magnitude smaller than the original values. This dramatic decrease indicated that the original sulphate values recorded were extremely high and inaccurate since they did not thermodynamically balance with the other cationic water quality parameters measured (i.e. Fe and Mn). This highlighted the experimental inaccuracies associated with the traditional analytical procedures used to measure sulphate concentrations in AMD and further served as experimental support for the need to conduct geochemical modelling as a data pre-cleaning step.

From Fig. S1a and Fig. S2a (which can be found in the Supplementary Information document along with Table S1 that gives the statistical values for each water quality parameter), several outliers were identified and removed to give the final clean distributions of each water quality parameter. The cleaned distributions (Fig. S1b and Fig. S2b) displayed a double hump behaviour which indicated two favoured values. This could be due to temperature variations and other geographic variations experienced over different seasons and the weather conditions where one value is favoured in hot, sunny conditions and other values in favoured in colder conditions (Chen & Jiang, 2012; Valente & Gomes, 2008). From Fig. S2 SUL and Fe have the same double hump shape which serves as experimental evidence for their extremely strong correlation and also indicated the presence of sulphide minerals such as pyrite (Valente & Gomes, 2008). Mn, TEMP and EC showed similar depressed hump shapes which indicated that these parameters were correlated to each other. TDS and pH had unique shapes which indicated that they were not dependant on any other water quality parameter.

**Fig. 1:**
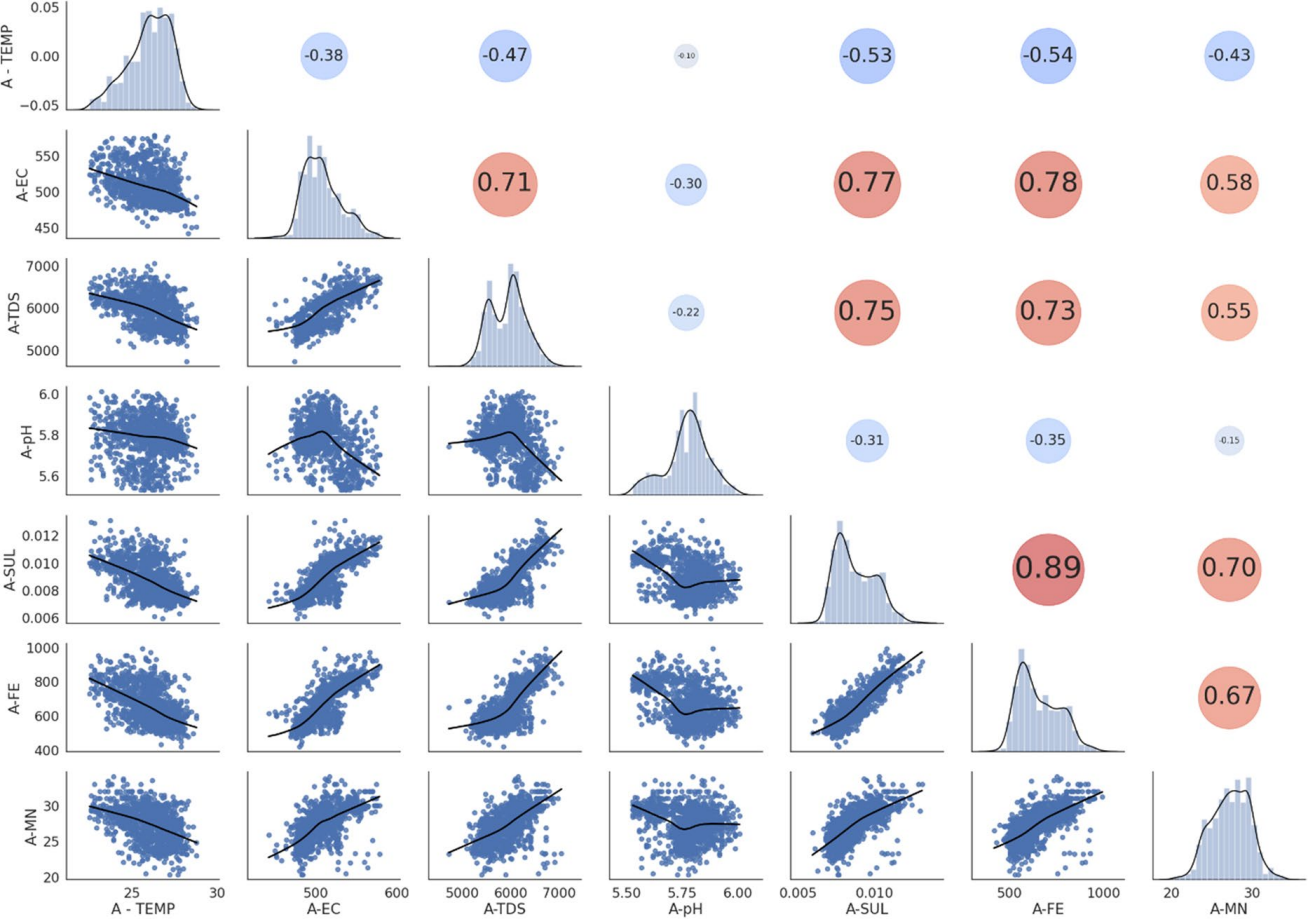
Scatter matrix (below main diagonal) and Pearson’s correlation matrix (above main diagonal) of Pump A indicate the interrelationships between water quality parameters. Diagonal histogram and density plots indicate the distribution of each parameter. For the correlation matrix, red circles are positive correlations, blue circles are negative correlations and larger circles indicate more strongly correlated variables

From the scatter matrix and correlation matrix (Fig. 1), it was found that TEMP and pH were negatively correlated (i.e. have an indirect relationship) with all other water quality parameters and that all other parameters were positively correlated (i.e. have a direct relationship) with each other. This indicated that there were indeed patterns present in the dataset and predictors were correlated to each other. The strongest negatively correlated variable was TEMP and this indicated that as temperature increased, all the other parameters decreased and this was noted as a significant finding since temperature is directly dependant on the season. One explanation for the negative correlation of TEMP with all other variables could be due to the energy required to break apart the metal sulphate bonds during the treatment process, as a result, this would lead to energy from neighbouring molecules being absorbed resulting in a decrease in temperature (Chen & Jiang, 2012). A practical implication of this is that the treatment plant was in fact functioning as desired and was successful in treating the AMD. Fe was seen to be the most strongly positively correlated variable (0.89) meaning that as Fe increased, so too did the other variables (besides temperature). Another explanation for the high Fe and SUL correlation could be due to the concentrated sulphuric acid used to leach uranium oxides as a by-product from gold mine slurry (Förstner & Wittmann, 1976). Iron pyrites present in this gold sludge react with the concentrated sulphuric acid and are discharged as AMD (Förstner & Wittmann, 1976). Since South Africa in particular has a strong history of operative and lucrative gold mines, a lot of gold-related sludge and, hence, concentrated sulphuric acid that reacted with pyrites would be present in the AMD.

**Fig. 2:**
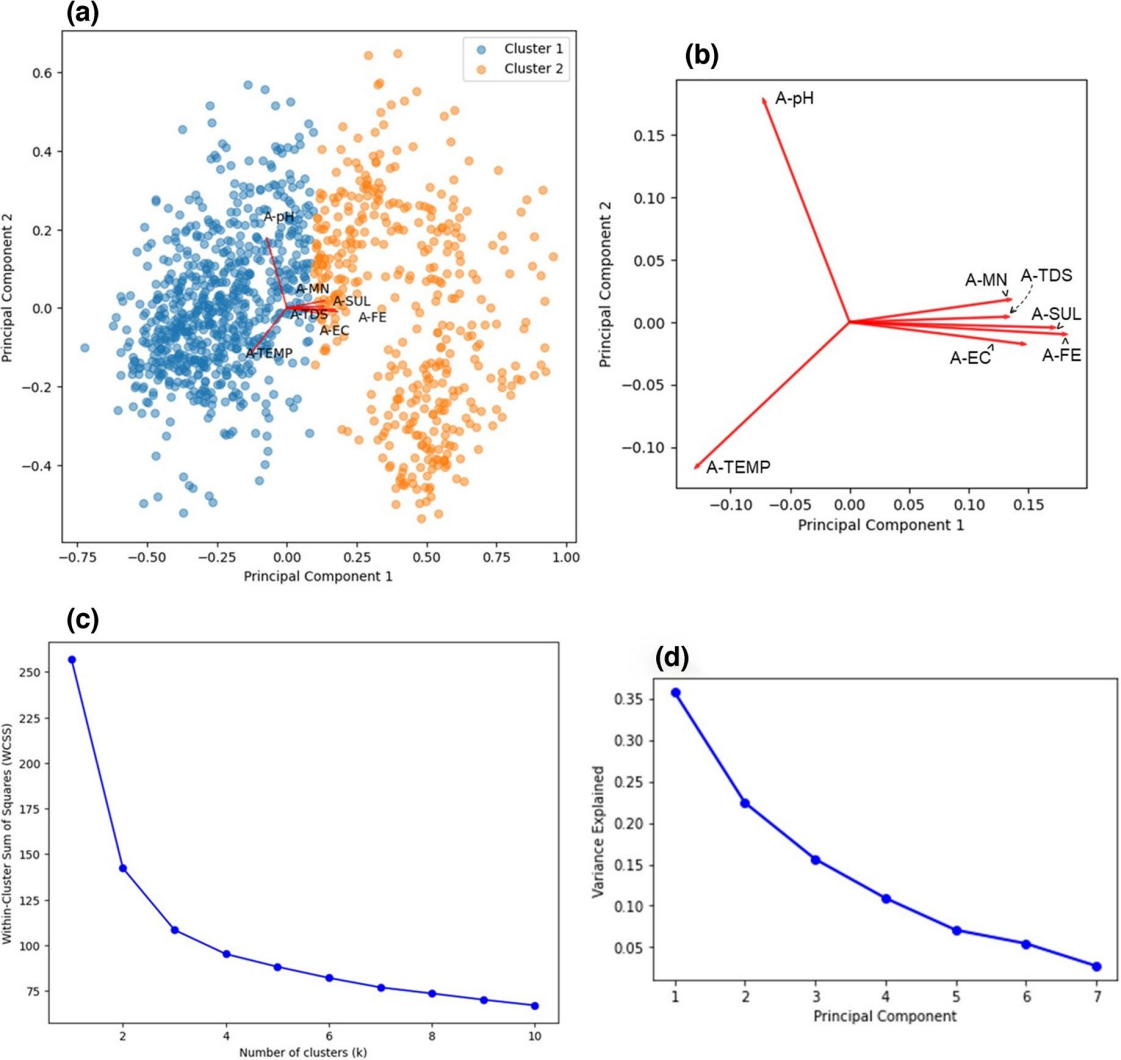
Dimensionality reduction and feature extraction results obtained from PCA for Pump A, **a** Bi-plot indicating the clustering of individual observations and its relation to the loadings plot. **b** Expanded view of the loadings plot indicating the relationship between parameters. **c** Elbow method plot indicating the optimal number of clusters. **d** Scree plot indicating the amount of variance explained by each principal component

Upon conducting PCA, from Fig. 2a–c, two clusters were observed in the bi-plot and this was consistent with the double hump behaviour seen in the density plots which gave further evidence for the temperature and seasonality (hot and cold environments) dependence of the water quality parameters. The loadings plot (Fig. 2b) revealed that A-MN, A-TDS, A-FE and A-EC were strongly related to A-SUL. From Fig. 2d, only two predictors were needed to explain 78% of the variance in the data and from RFE these variables were A-TDS and A-FE. TDS has been found to be strongly correlated to heavy metals since it is a measure of the inorganic material content (dissolved metals, calcium, magnesium, sulphates, etc.) present in the water (Hatar et al., 2013), and so it would account for a lot of variability in the data. As discussed, Fe was strongly correlated to SUL, and since Pyrite is one of the most abundant sulphide minerals on the planet (Johnson & Hallberg, 2005b) and is possibly one of the main constituents in Pump A’s AMD since Fe is one of the principal components.

**Fig. 3:**
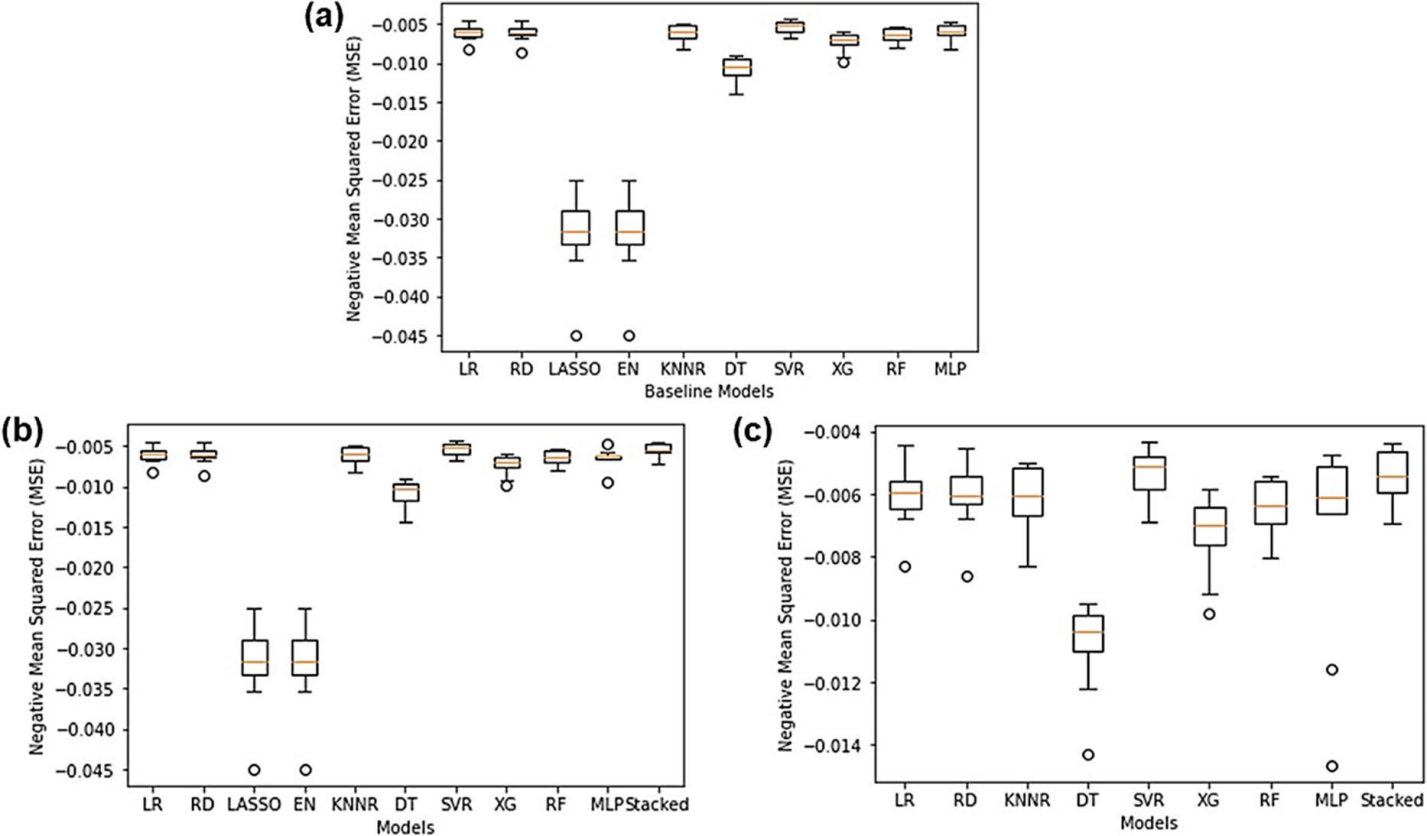
Regression algorithm NMSE comparison for Pump A showing **a** all individual baseline models, **b** all models and a stacking regressor containing all the models and **c** all well-performing models and a stacking regressor containing all the best-performing models

As seen in Fig. 3a, upon comparing individual machine learning algorithms it was found that the SVR performed the best (NMSE: − 0.00532) whilst LASSO (NMSE: − 0.03217) and EN (NMSE: − 0.03217) performed the worst. All other models performed around the same level. Since there existed a high degree of correlation between predictors, LASSO performed poorly since there was high bias and multicollinearity present in the data and LASSO only selects on variable and sets the rest to zero (Muthukrishnan & Rohini, 2016). Since EN essentially combines LASSO and RD, the poorly performing LASSO could have tainted and ruined the predictive accuracy of EN due to its inability to handle multicollinearity. The SVR is quite tolerant to outliers since slack can be added to the margin and works well on smaller datasets (Awad & Khanna, 2015; F. Zhang & O’Donnell, 2020), therefore, since Pump A’s dataset only had 1107 rows of data the SVR showed good predictive accuracies. The exact NMSE values for all models can be found in Table S2 in the “Supplementary Information” document.

As seen in Fig. 3b, when combining all the baseline models into a single stacking regressor, the performance of the stacking regressor (NMSE: − 0.00544) was better than that of the individual models except for the SVR. This showed that combining the predictive accuracies of each of the models did improve the overall predictive performance of the model; however, the SVR still performed this best. This can be attributed to the SVR being tolerant towards outliers and appropriate for smaller datasets, whereas some of the other models combined in the stacking regressor were sensitive to outliers and required large datasets to be able to make accurate predictions. This was in accordance with two other studies which reported that SVR outperformed ensemble methods due to its outlier tolerance (Corte et al., 2020; García-Gutiérrez et al., 2015; Y. Zhang et al., 2022). However, it is still worth nothing that the stacking regressor that included the LASSO and EN still performed exceptionally well having included them due to combining the “wisdom of the group”.

It was then decided to remove the bad-performing models and create a new stacking regressor that only contained the best-performing models to determine the effect of excluding the bad-performing models (LASSO and EN). As seen in Fig. 3c, when combining only the best-performing models into a single stacking regressor the predictive accuracy of the stacking regressor (NMSE: − 0.00546) did not drastically improve and the SVR still performed the best. Thus, it was concluded that including worse-performing models (LASSO and EN) in the stacking regressor had no effect on its predictive performance as long as the well-performing models were present (SVR).

**Fig. 4:**
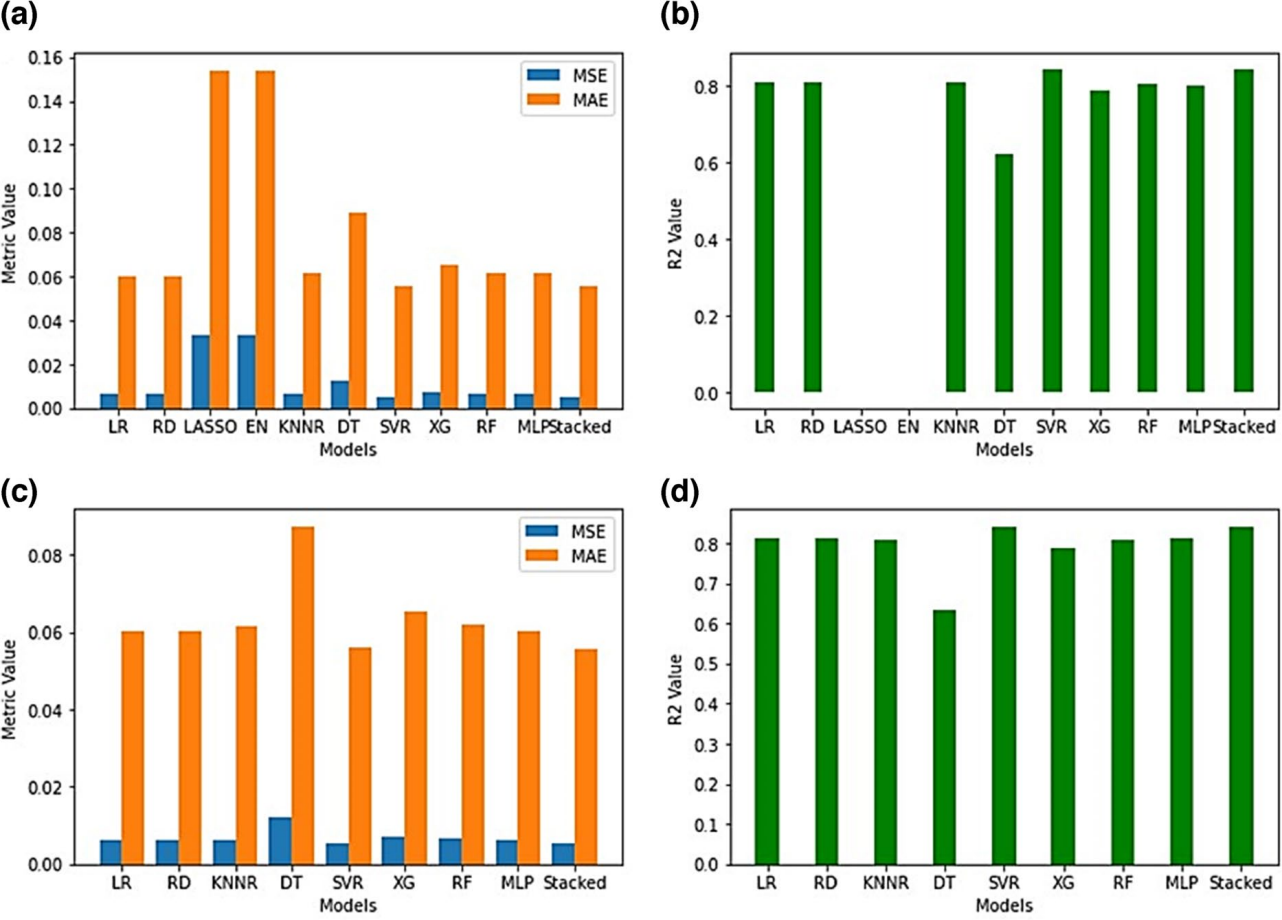
Testing statistics (MSE, MAE and *R*^2^) accuracy comparison of regression models trained on Pump A. **a**, **b** Stacking regressor using all models. **c**, **d** Stacking regressor using only the best-performing models

Upon testing the models (Fig. 4a and b) each of the baseline models and the stacking regressor containing all the models did achieve low MSE, low MAE and high *R*^2^ values besides the LASSO and EN which achieved high MSE, high MAE and extremely low *R*^2^ values (MSE:0.033252, MAE:0.153985 and *R*^2^: − 0.000069 respectively). This served as further experimental evidence that the LASSO and EN were not the optimal models to be used to predict sulphate levels in Central Rand Pump A. However, during testing it was found that the stacking regressor performed the best (MSE:0.005252, MSE:0.055598 and *R*^2^:0.842041) and this could be attributed to the reduced overfitting of the stacking regressor as opposed to the SVR (MSE:0.005275, MAE:0.055935 and *R*^2^:0.841351) since the stacking regressor combined the individual models, thereby, averaging out the individual biases and variances. As observed in Fig. 4c and d, when testing the stacking regressor using only the best-performing models (MSE:0.005335, MAE:0.055679, *R*^2^:0.839538), it was no longer clear as to whether the SVR or the stacking regressor performed better. The MSE, MAE and *R*^2^ values also did not drastically improve from the stacking regressor that contained all the models. This served as further experimental evidence that the inclusion of worse-performing models in the stacking regressor had no effect on its overall performance in Pump A. The exact MSE, MAE and *R*^2^ values for all models can be found in Table S2 in the “Supplementary Information” document.

### Pump B

The original sulphate levels pertaining to Central Rand Pump B were in the range of 3000–4000 mg/L; however, after geochemical modelling these values, now lied in the range of 0.002–0.0015 mg/L and this decrease was consistent with that seen in Pump A. Many outliers were present in the dataset as observed in Fig. S3a and Fig. S4a which were subsequently removed. After outlier removal, from Fig. S3b and Fig. S4b, the same double hump behaviour was seen for Pump B and this provided further evidence for the seasonal and temperature dependence of the water quality parameters. Descriptive statistics for each water quality parameter can be found in Table S3.

**Fig. 5:**
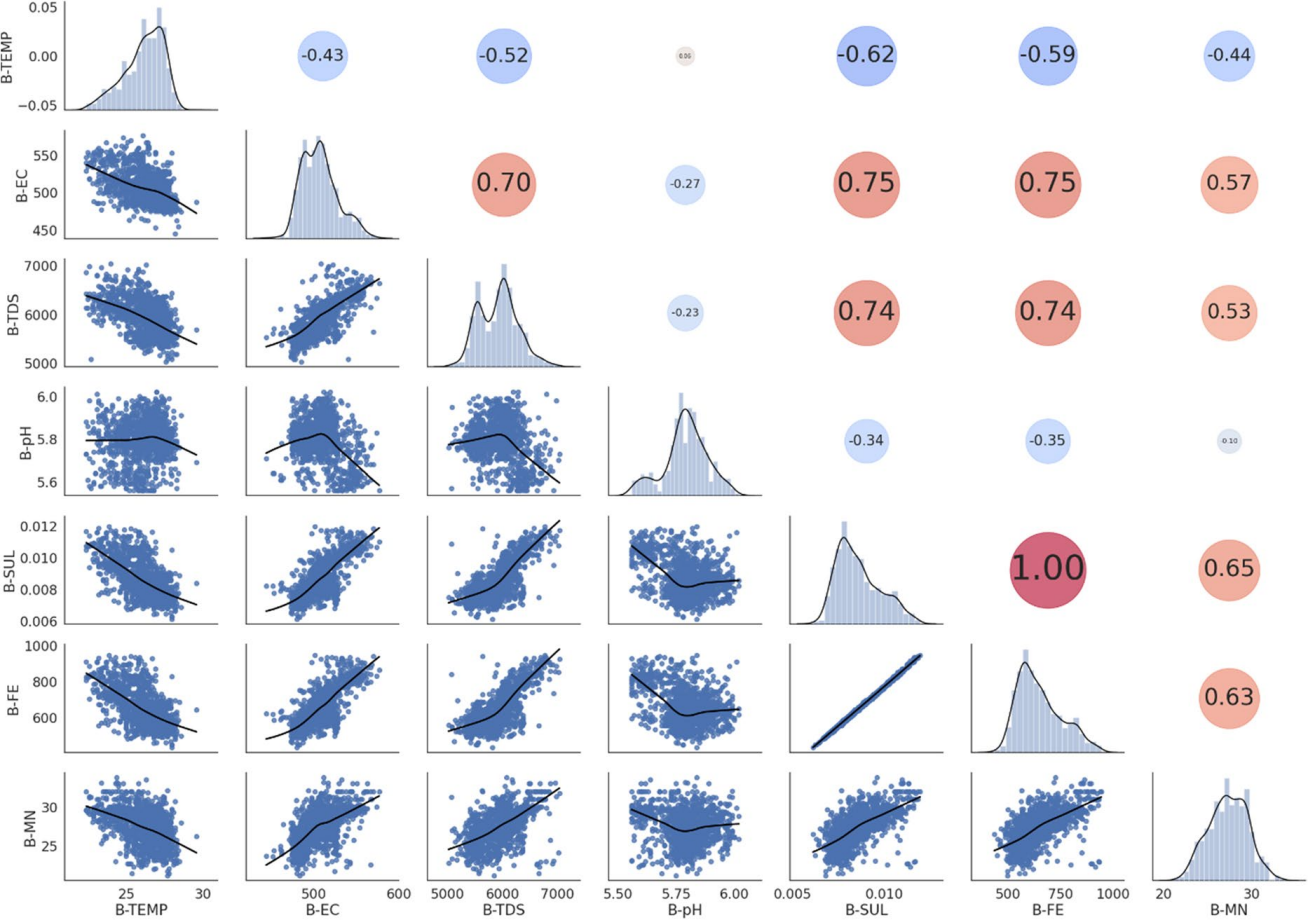
Scatter matrix (below main diagonal) and Pearson’s correlation matrix (above main diagonal) of Pump B indicate the interrelationships between water quality parameters. Diagonal histogram and density plots indicate the distribution of each parameter. For the correlation matrix, red circles are positive correlations, blue circles are negative correlations and larger circles indicate more strongly correlated variables

From Fig. 5, the same trends observed for Pump A were observed for Pump B. Temperature was still negatively correlated to all other water quality parameters, iron was perfectly positively correlated with sulphates since it attained a correlation of 1.0 and this was strong evidence for the presence of dissolved pyrites as the main constituent in AMD. EC and TDS were even more positively correlated with sulphates than Pump A and pH showed extremely little correlation to all other water quality parameters.

**Fig. 6:**
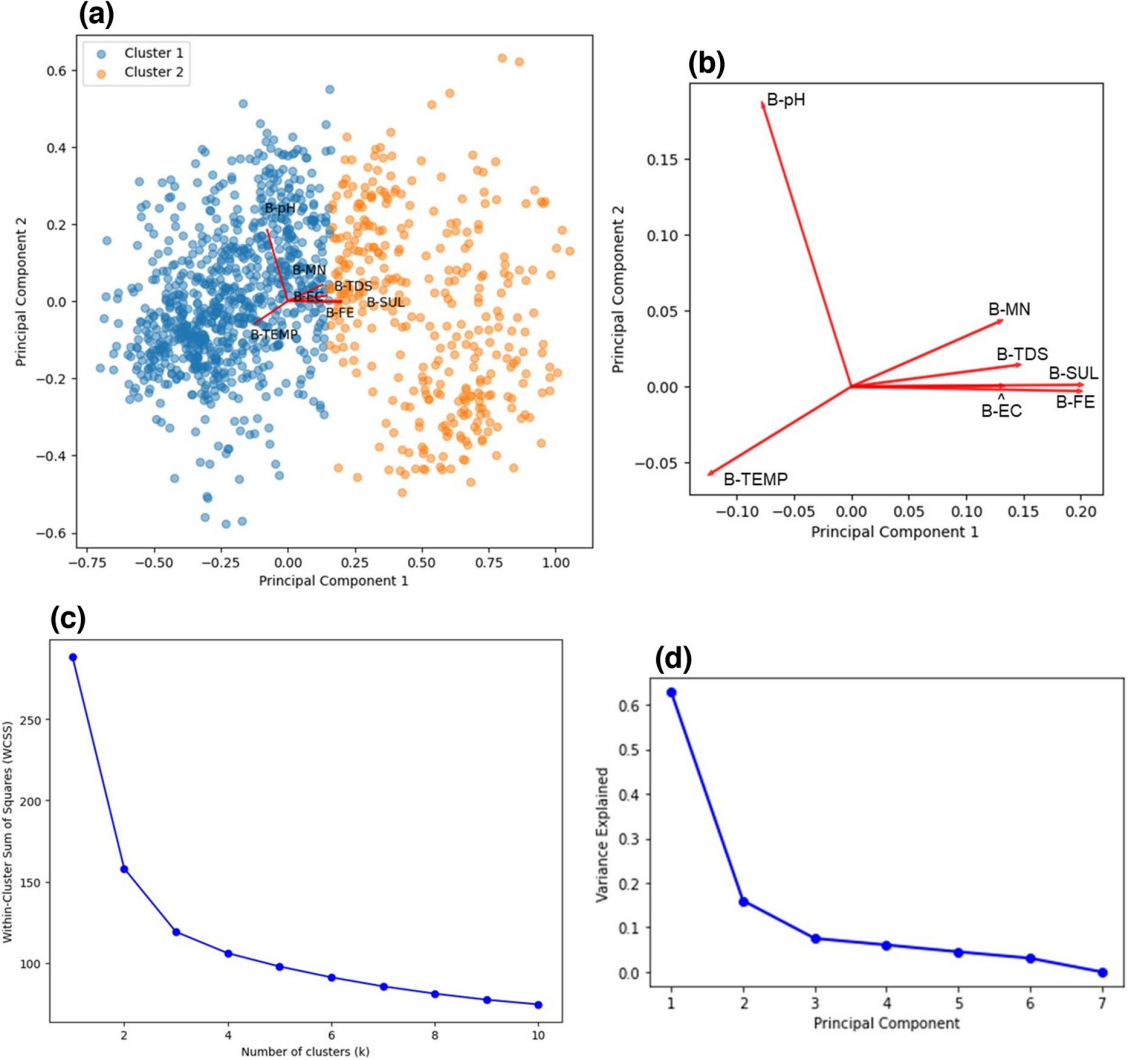
Dimensionality reduction and feature extraction results obtained from PCA for Pump B. **a** Bi-plot indicating the clustering of individual observations and its relation to the loadings plot. **b** Expanded view of the loadings plot indicating the relationship between parameters. **c** Elbow method plot indicating the optimal number of clusters. **d** Scree plot indicating the amount of variance explained by each principal component

From the bi-plot (Fig. 6a and c) two clusters were again observed for Pump B and from the loadings plot (Fig. 6b) it was observed that the same parameters (B-MN, B-TDS, B-FE and B-EC) were highly correlated to B-SUL, which agreed with what was observed for Pump A. The Scree plot (Fig. 6d) revealed that two components, viz, B-TEMP and B-FE, were needed to explain 79% of the variance in the data. This agreed with the trends observed in the scatter matrix and the correlation matrix (Fig. 5) for Pump B that showed the temperature having the strongest negative correlation (− 0.62) with sulphate and iron having the strongest positive correlation (1.0) with sulphate.

**Fig. 7:**
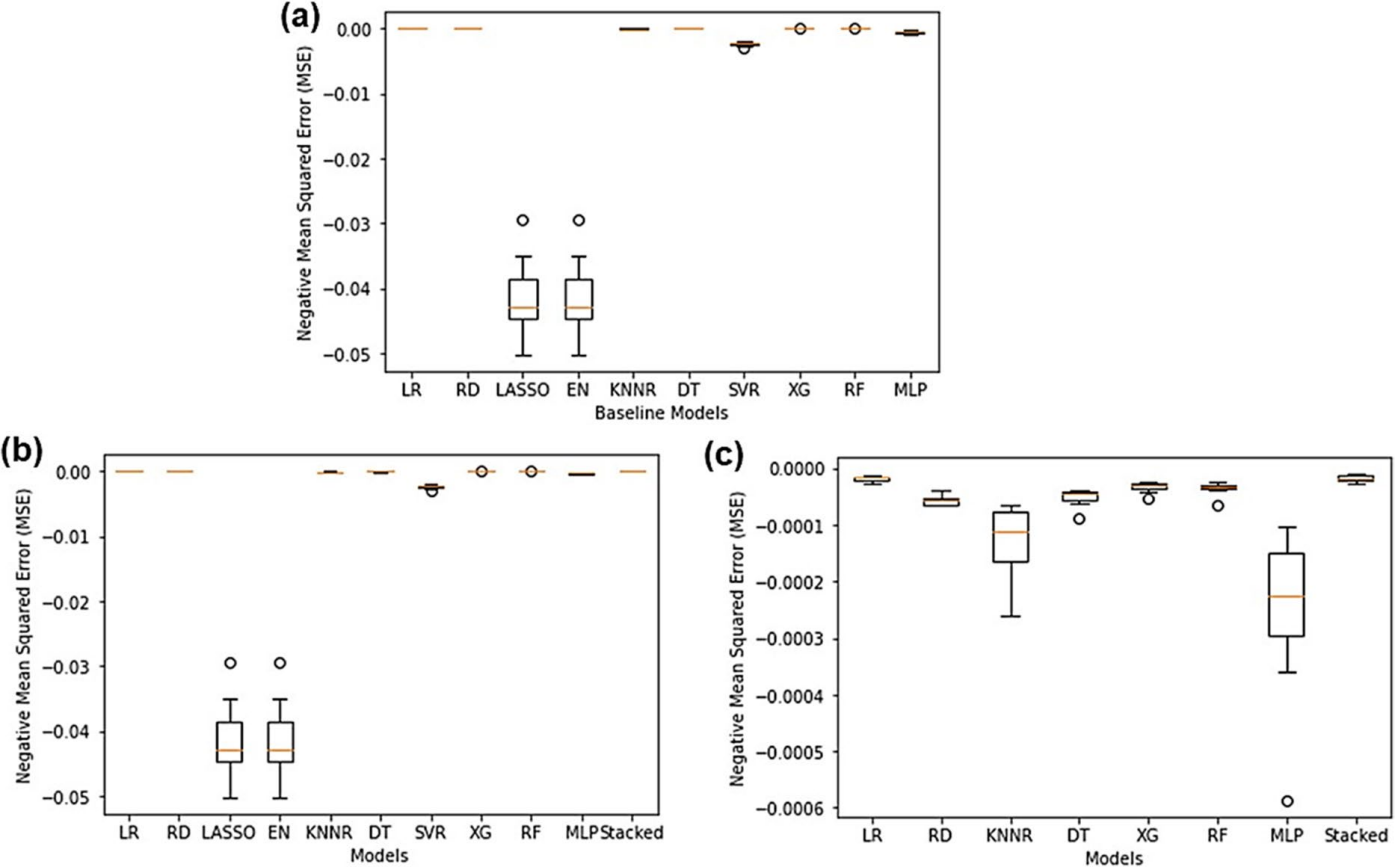
Regression algorithm NMSE comparison for Pump B showing **a** all individual baseline models, **b** all models and a stacking regressor containing all the models and **c** all good-performing models and a stacking regressor containing all the best-performing models

As seen in Fig. 7a, the LASSO and EN (NMSE: − 0.04149) once again were the worst-performing models due to their inability to handle multicollinearity. The best baseline model was LR (NMSE: − 0.000018) and this correlated with what was observed in the scatter and correlation matrices since iron was perfectly linearly related to sulphates. Thus, LR was mathematically the perfect algorithm to use for predictions.

When combining all the models into a stacking regressor, as seen in Fig. 7b, the stacking regressor (NMSE: − 0.000016) outperformed LR which provided experimental evidence for the enhanced predictive power of ensemble models compared to single baseline models. Even though the stacking regressor contained poorly performing models such as LASSO and EN, it was still able to harness the combined predictive power of all the other models and was the best-performing model as a result thereof. When combining only the best-performing models into a stacking regressor, as seen in Fig. 7c, the performance of the stacking regressor did not improve (NMSE: − 0.000016) compared to when all the models were combined. This was in accordance with what was observed for Pump A and provided further experimental evidence to the fact that including poorly performing models in a stacking regressor has no effect on its overall performance as long as the good-performing models were present as well.

**Fig. 8:**
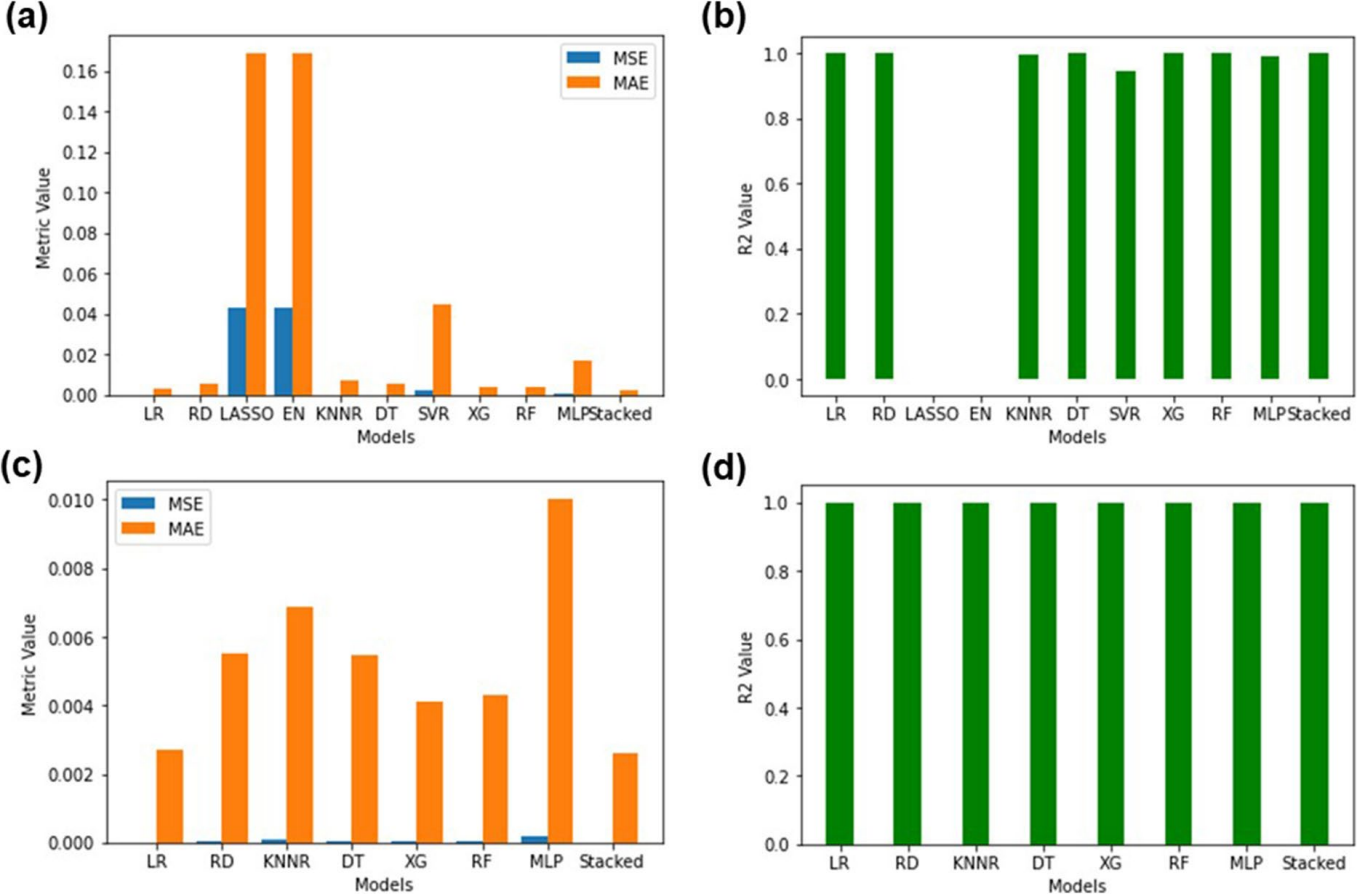
Testing statistics (MSE, MAE and *R*^2^) accuracy comparison of regression models trained on Pump B. **a**, **b** Stacking regressor using all models. **c**, **d** Stacking regressor using only the best-performing models

Upon testing the models, as observed in Fig. 8a and b, LASSO and EN (MSE:0.043197, MAE:0.169071 and *R*^2^: − 0.000104) still performed the worst and the stacked ensemble using all the models performed the best (MSE:0.000011, MAE:0.002656 and *R*^2^:0.999741). Noticeably LASSO and EN had negative *R*^2^ values which indicated that the predictions made using these models were worse than simply using the mean value to replace missing values. From Fig. 8c and d, the stacking regressor using only good models showed phenomenal performance (MSE:0.000011, MAE:0.002617 and *R*^2^:0.999737) and did show a slight improvement compared to that of the stacking regressor that utilized all the models. Individual model performance metrics can be found in Table S4.

### Treated Water

A dramatic decrease in the sulphate levels after geochemical modelling was once again observed for the Treated Water since the values decreased from 2000–3000 to 0.020–0.035 mg/L. The Treated Water also contained a lot more outliers (Fig. S5a and Fig. S6a) than Pump A and Pump B, and after outlier removal (Fig. S5b and Fig. S6b) the distributions for each of the water quality parameters did significantly improve; however, one unusual distribution was found for Fe. Instead of a normal distribution, Fe showed an almost smooth decrease with two missing bins which stood in stark contrast to the Fe distributions observed for Pump A and Pump B. With regard to all other water quality parameters, only single mean values were observed instead of the double hump behaviour as seen for Pump A and Pump B. These changes in distributions were indicative of the proper operation of the AMD treatment plant since all the water quality parameters were significantly altered after the treatment process which indicates a change in the overall water chemistry of the treatment process. Most of the metals such as Fe and MN would have been removed as sludge during the treatment process and due to the addition of basic neutralizing agents the pH of the Treated Water was expected to increase, and indeed it did increase to 8.71 ± 0.179. Descriptive statistics for each water quality parameter for the Treated Water can be found in Table S5.

**Fig. 9:**
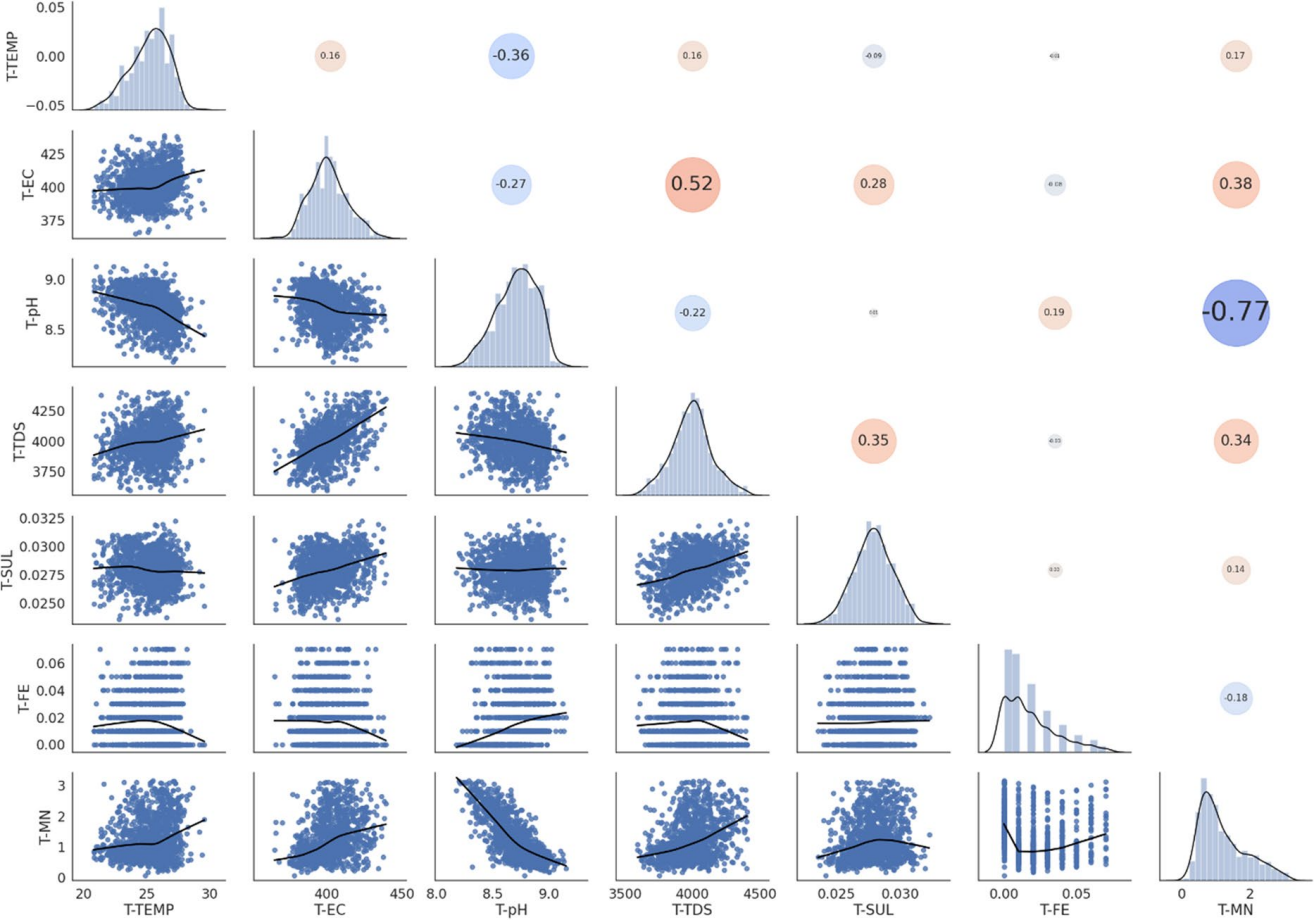
Scatter matrix (below main diagonal) and Pearson’s correlation matrix (above main diagonal) of Treated Water indicate the interrelationships between water quality parameters. Diagonal histogram and density plots indicate the distribution of each parameter. For the correlation matrix, red circles are positive correlations, blue circles are negative correlations and larger circles indicate more strongly correlated variables

As observed in Fig. 9, there was almost no inter-relationships and correlations between the water quality parameters in the Treated Water. Instead of linear relations, values were clustered around certain points which translated to a near zero correlation. This immediately hinted at the lack of inherent patterns present in the Treated Water dataset. The only strong correlation was found to be between pH and MN (− 0.77) which indicated that as pH increased, the amount of manganese decreased. Noticeably, the distribution of Fe was in distinct bins instead of a cluster or trendline, and this was incredibly unusual. This led to almost no correlation between Fe and all other water quality parameters which was extremely strange due to Fe showing such a high correlation to SUL in Pump A and Pump B.

Due to basic neutralizing agents being added during the treatment process, in the process any Fe bound to SUL was liberated which dramatically altered the Fe distribution and increased SUL concentrations. Therefore, since pH is related to SUL, an increase in the amount of basic neutralizing agents added increases the pH which in turn liberates more SUL. Mn may not have been effectively removed from the AMD during the treatment process and could have still been present in the treated effluent waters as manganese (II) sulphate which could possibly account for its correlation to SUL.

**Fig. 10:**
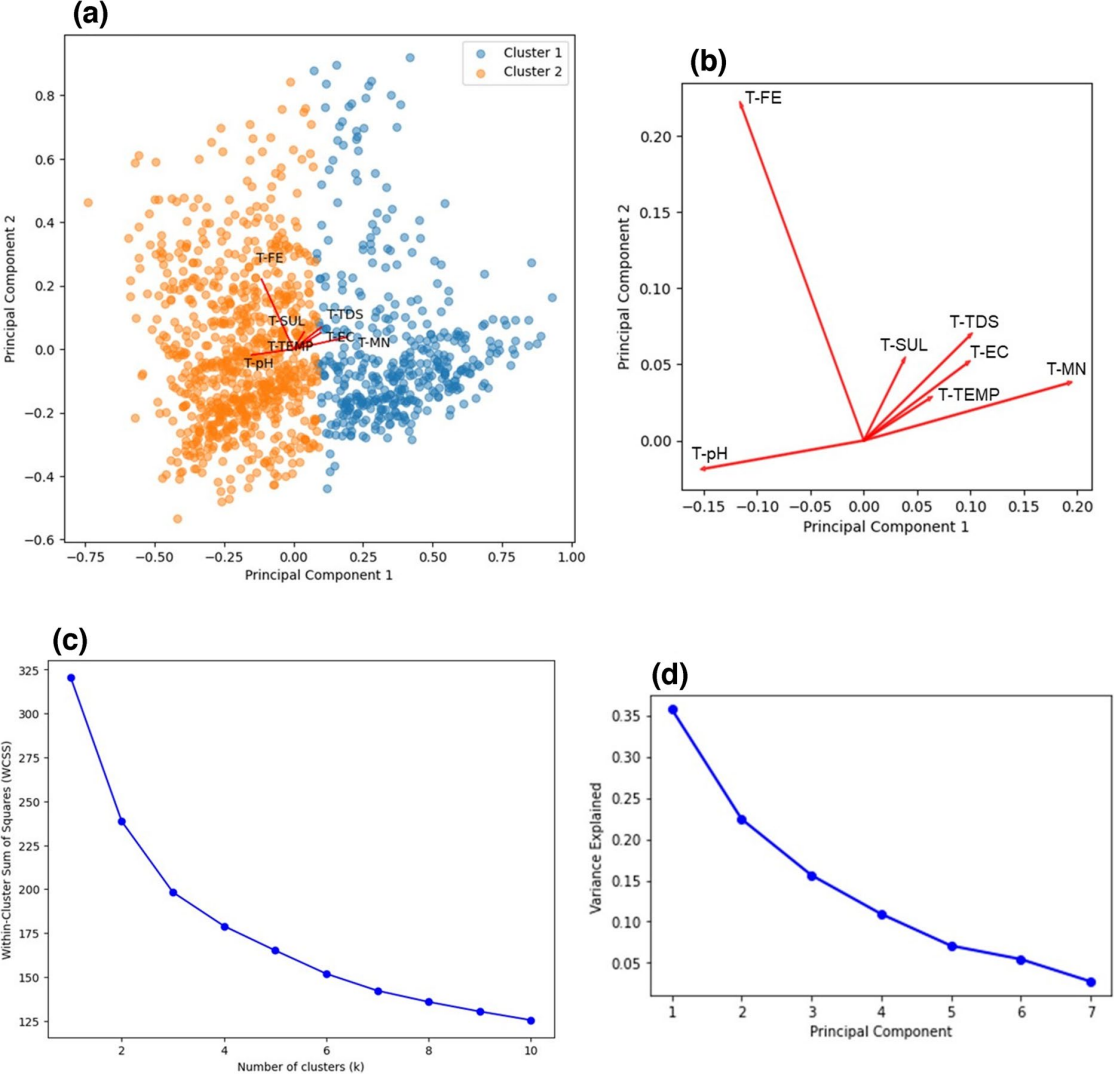
Dimensionality reduction and feature extraction results obtained from PCA for the Treated Water. **a** Bi-plot indicating the clustering of individual observations and its relation to the loadings plot. **b** Expanded view of the loadings plot indicating the relationship between parameters. **c** Elbow method plot indicating the optimal number of clusters. **d** Scree plot indicating the amount of variance explained by each principal component

The bi-plot (Fig. 10a–c) once again revealed two clusters which gave further evidence for the temperature dependence of the water quality parameter values. However, the loadings plot (Fig. 10b) now indicated that T-FE was no longer related to T-SUL, instead T-MN, T-TDS, T-EC, T-TEMP and T-MN were strongly related to SUL which agreed with the trend observed in the scatter and correlation matrices (Fig. 9). From the scree plot (Fig. 10d), only three components were needed to explain 74% of the variance in the data and these predictors were T-pH, T-TDS and T-MN. Due to the low correlation observed between variables, more predictors were needed in the Treated Water dataset (three predictors) as compared to Pump A and Pump B (two predictors). Noticeably, Fe was not selected as a predictor for the Treated Water as opposed to that of Pump A and Pump B and this was in accordance with its unusual distribution and low correlation to SUL.

**Fig. 11:**
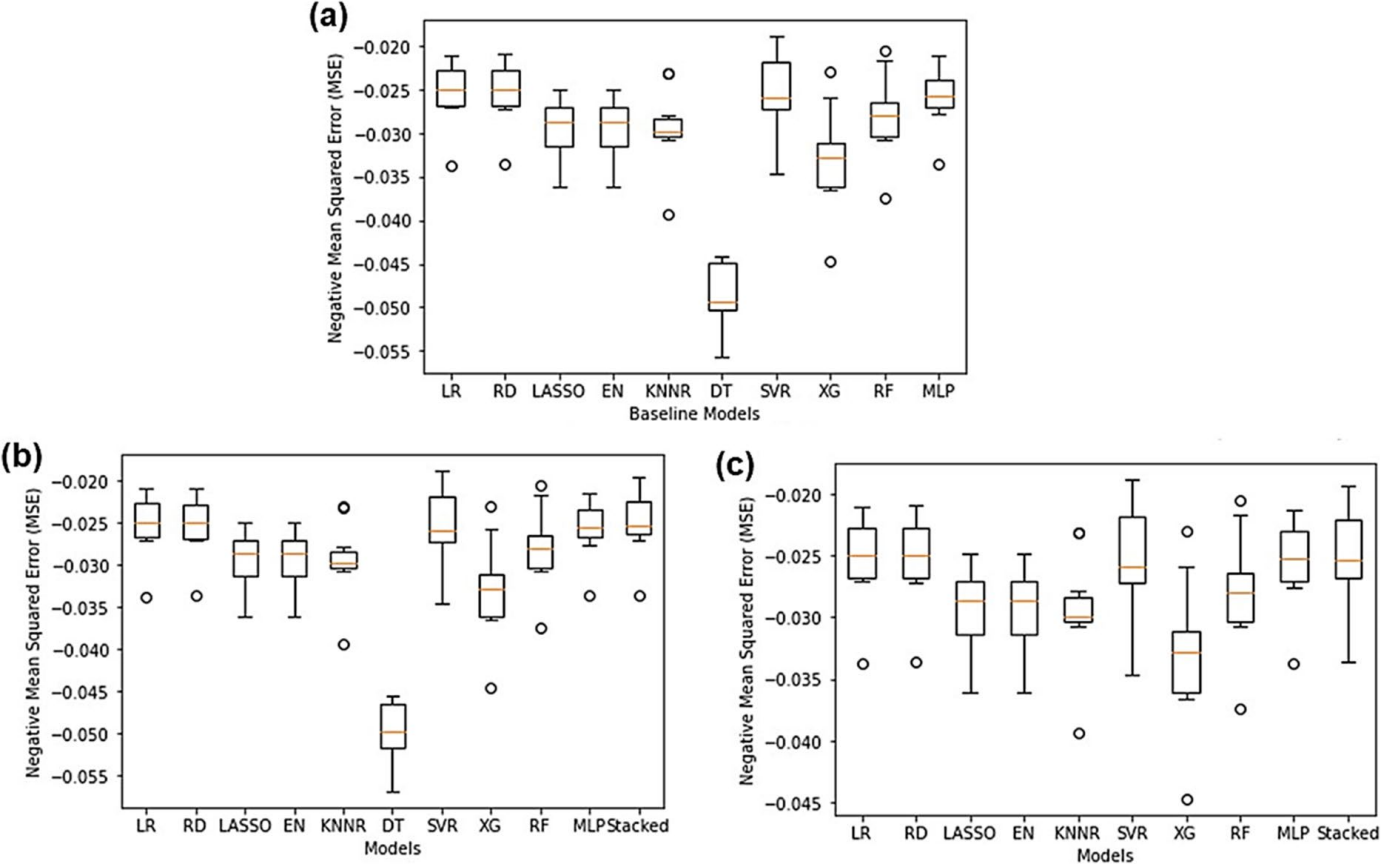
Regression algorithm NMSE comparison for Treated Water showing **a** all individual baseline models, **b** all models and a stacking regressor containing all the models and **c** all good-performing models and a stacking regressor containing all the best-performing models

From Fig. 11a, the DT was the worst-performing baseline model (NMSE: − 0.048447) and the SVR once again was the best-performing model (NMSE: − 0.025181). DT are extremely susceptible to small variations in the data (Pekel, 2020), and due to the highly uncorrelated nature of the Treated Water data a lot of variations were present in it and hence, the DT performed poorly. SVR performed well due to its ability to handle outliers and was suitable to be used on the Treated Water dataset since it was small and only consisted of 1197 rows of data.

As seen in Fig. 11b, the stacked ensemble model (which combined all the baseline models) did outperform all other models (NMSE: − 0.024949) since it was able to combine the predictive accuracy of all the baseline models and learn from their mistakes. From Fig. 11c, when only combining the best models into a stacking regressor, the stacking regressor actually performed worse (NMSE: − 0.025005) than the stacking regressor that used all the models. A possible explanation for this is that stacking regressors not only combine predictive accuracies of the level 0 models, but they also learn from the level 0 model mistakes. Thus, when omitting the bad-performing models from the stacking regressor, there was actually less knowledge to learn from since not many mistakes were made and so further improvements could not be made as to what not to do or what to improve on. This was a remarkable discovery since it implied that including “failed” or “poorly performing” models in stacking regressors improved their overall performance for the Treated Water.

**Fig. 12:**
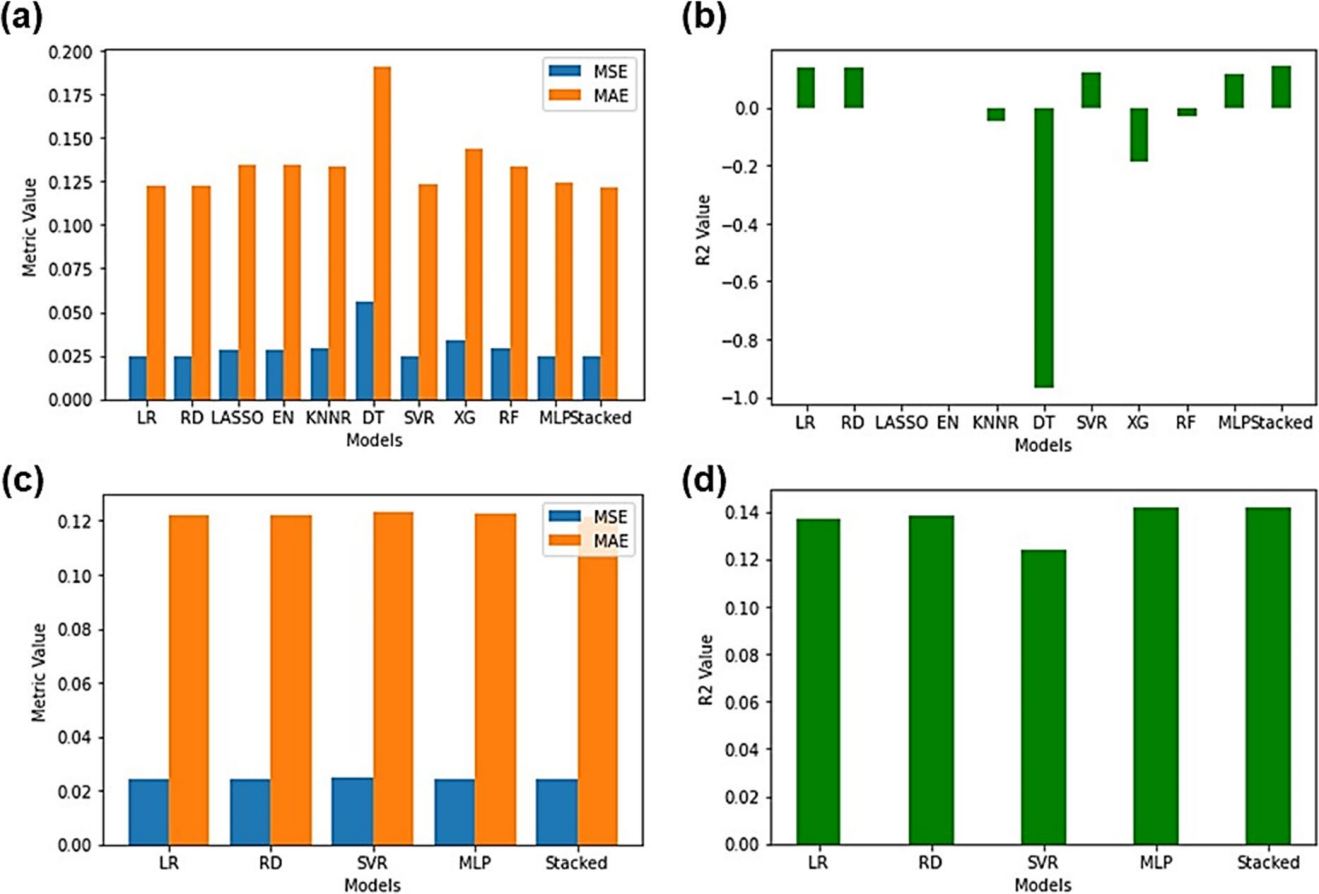
Testing statistics (MSE, MAE and *R*^2^) accuracy comparison of regression models trained on Treated Water. **a**, **b** Stacking regressor using all models. **c**, **d** Stacking regressor using only the best-performing models

As seen in Fig. 12a–d, the DT once again performed the worst during testing (MSE:0.055957, MAE:0.191006 and *R*^2^: − 0.967734) and the best-performing model was the stacking regressor that combined all the models (MSE:0.024362, MAE:0.121571 and *R*^2^:0.143315). However, the *R*^2^ values obtained for the Treated Water were dismal since the highest value that could possibly be obtained by even the best-performing model was 0.14. Models such as LASSO, EN, KNNR, DT, XG and RF all produced negative *R*^2^ values which indicated that the inherent unusual data distributions were the cause of the poor model performance. Any model that achieved a negative *R*^2^ value indicated that the model performed worse at predicting a sulphate value than just replacing the missing value with the mean sulphate value. Upon comparison to Pump A and Pump B, this stood in stark contrast since models trained on Pump A and Pump B’s data achieved phenomenal *R*^2^ values. A possible explanation for the poor *R*^2^ observed with the Treated Water could be due to the treatment process itself. Due to many neutralizing agents being added and the water going through many physically intensive processes, any chemical relationships between the species present in the water would be drastically altered or even removed. This could have resulted in the extremely low correlations and unusual distributions observed. Individual testing metric values for each model can be found in Table S6.

Many attempts were made to improve the *R*^2^ value such as removing Fe completely from the dataset due to its unusual distribution and applying different transforms to the data such as a “StandardScaler” (which transforms the predictors to have a mean of 1 and standard deviation of 0) and a “Normalizer” (which transform each row to have a length of 1). Unfortunately, none of these were successful; however, as observed in Table 2, it was discovered that the number, type of predictors and percentage of variance explained was heavily dependent on the type of transform applied.

**Table 2:** Dependence of important features determined by PCA and RFE on type of data transform applied to the Treated Water data

Type of transform applied	Most important features	Variance explained (%)
Removed T-FE	T-TEMP, T-EC and T-TDS	79
StandardScaler	T-EC, T-pH, T-TDS and T-MN	81
Normalizer	T-FE	98
Removed T-FE and Normalizer	T-pH	98

### Implications and limitations of the findings

This study provides evidence for the use of different stacking ensemble machine learning regressors to predict water quality parameters in AMD due to their robustness and enhanced predictive accuracies, a topic that has previously gone unreported.

Due to the high accuracy obtained from the Pump B SE-ML regressor that combined only the best-performing models, this stacked model is suitable for transfer learning (TL) to impute missing sulphate levels in other AMD treatment plants. TL is an important approach to use in augmenting data in sparse or scanty datasets (Weiss et al., 2016). As such, its success is dependent on the initial dataset being accurate with low uncertainties (Niu et al., 2020). It should be noted though here that TL works on similar datasets, in this case similar chemistry of the water. This is because, as with any ML model, bias becomes an inherent property depending on the dataset used for training and validation (Kouw & Loog, 2019). In this study, the implication of this was apparent in the sulphate levels that were found to greatly vary between the AMD feed and treated AMD, which indicated the change in water chemistry following the treatment process. As such, the two datasets have to be modelled separately and have to be used for respective similar sparse datasets during TL. It is important to underscore the fact that the quality of the models is dependent on the quality of the data used (Kouw & Loog, 2019). Analytical uncertainties, spurious and generally poorly analysed water parameters can present inconclusive and incoherent ML models. It is interesting that such challenges can be deduced from ML models, implying that modelling can be a useful tool in assessing the quality of laboratory analyses and providing insights into areas requiring attention and improvement. As indicated previously, these findings also have a bearing on further work (e.g. hydrochemical modelling and experimental) aimed at using the predicted water chemistry to precipitate out valuable resources such as elemental sulphur, sulphates, ochres and important metals such as copper, nickel, cobalt, rare earth elements, uranium and gold.

Potential sources of bias could also come in from only one treatment plant dataset being used in this study and hence, only obtaining water quality results from one laboratory. In future, several AMD treatment plant datasets should be sourced in order to increase the variety present in the dataset and remove any inherent bias associated with only using dataset from a single treatment plant. Whilst this study is currently limited by data obtained from only one AMD treatment plant, future work also includes sourcing other Treated Water data from a different AMD treatment plant in order to verify if the lack of inherent patterns within the Treated Water dataset was due to the treatment process itself. Future work also includes using the trained SE-ML regressor for predicting sulphate levels in other AMD treatment plants.

## Conclusion

Individual (LR, RD, LASSO, EN, KNNR, SVR, DT and MLP) and ensemble (RF, XG, SE-ML) regression models were successfully trained and tested in order to predict sulphate levels in an Acid Mine Drainage treatment plant in Johannesburg, South Africa. The untreated AMD “Pump B” dataset produced the best-performing model, which was a stacking regressor that combined the best models namely LR, RD, KNNR, DT, XG, RF and MLP as the level 0 models and another LR as the level 1 model which performed phenomenally well and achieved MSE of 0.000011, MAE of 0.002617 and *R*^2^ of 0.999737 in under 2 minutes. Ensemble methods (bagging, boosting and stacking) did outperform individual models. Both SE-ML architectures showed a far greater accuracy than the rest of the models which proved that adopting SE-ML architectures for water quality prediction provides a more accurate approach. Surprisingly, SE-ML using only the best models in level 0 did not significantly outperform SE-ML that used all the models in level 0. This indicated that including poor-performing models in level 0 in a stacking regressor had no effect on its overall performance as long as the good-performing models were present in level 0.

Temperature (°C), total dissolved solids (mg/L) and, most importantly, iron (mg/L) were found to be the most important and most correlated values that were needed to predict sulphate levels with the strong iron-sulphate correlations indicating the presence of dissolved pyrites. Water quality parameter levels were found to be highly dependent on a variety of factors such as weather, season and sampling location. The Treated Water chemistry was significantly different to that of the untreated AMD due to the addition of basic neutralizing agents and the various physical processes it goes through during the treatment process, and as such, there were no inherent patterns in the Treated Water dataset which led to poor model performance (*R*^2^:0.1). Geochemical modelling using PHREEQC was found to be an important data-cleaning step in order to identify and remove outliers.

This study highlighted the ease of use, time-saving nature and cost-effectiveness of implementing individual and ensemble ML regression algorithms as a pre-cursor to providing a data for further experimentation and modelling to remediate AMD and extract valuable by-products (e.g. octathiocane), thus creating a sustainable circular economy.

### Supplementary Information

Below is the link to the electronic supplementary material.

Supplementary file1 (PDF 1001 KB)

## Data Availability

The dataset used for this work forms part of an ongoing study and is not available for public use.

## Code availability

The custom code used can be made available upon request.
