# Supplementary material for: Comparison of individual and ensemble machine learning models for prediction of sulphate levels in untreated and treated Acid Mine Drainage
Source: Environ Monit Assess. 2024 Mar 2;196(4):332. doi: 10.1007/s10661-024-12467-8 (PMC10907470; doi:10.1007/s10661-024-12467-8)
Supplement: Supplementary file 1 — Supplementary file1 (PDF 1001 KB) [file 10661_2024_12467_MOESM1_ESM.pdf]

Environmental Monitoring and Assessment

Comparison of individual and ensemble machine learning models for prediction of sulphate levels in untreated and treated Acid Mine Drainage

SUPPLEMENTARY INFORMATION

Taskeen Hasrod[1], Yannick B Nuapia[2], Hlanganani Tutu[1]*

[1]*Molecular Sciences Institute, School of Chemistry, University of the Witwatersrand, Private Bag X3, Johannesburg 2050, South Africa;*

[2]*Pharmacy Department, School of Healthcare Sciences, University of Limpopo, Turfloop Campus, 0727, Polokwane, South Africa.*

[1]**Corresponding author: Molecular Sciences Institute, School of Chemistry, University of the Witwatersrand, Private Bag X3, Johannesburg, 2050, South Africa, Email: hlanganani.tutu@wits.ac.za*

PUMP A

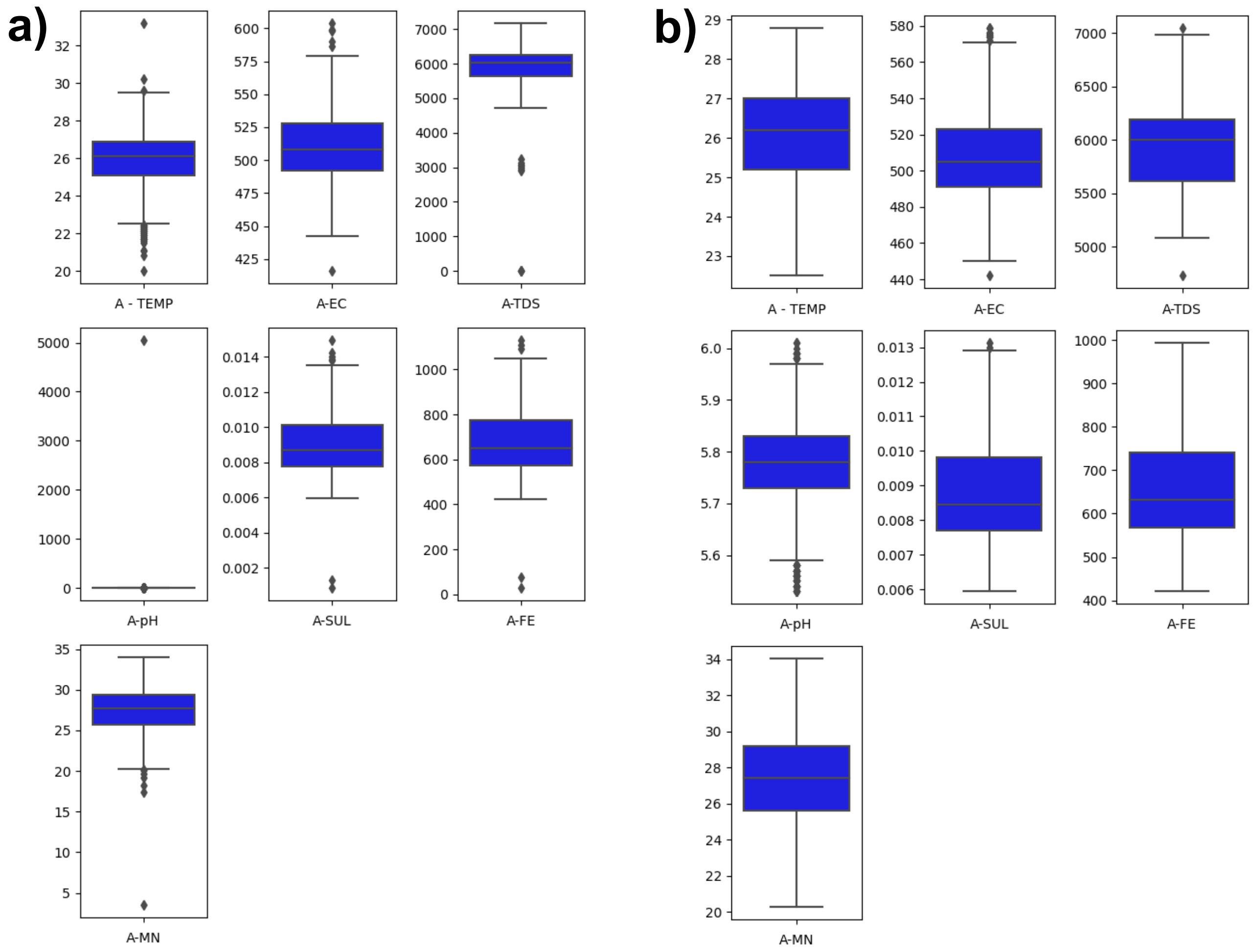

Fig. S1 *Box and whisker diagrams of Pump A indicating its statistical distribution a) Prior to outlier removal and b) After outlier removal*

*hlanganani.tutu@wits.ac.za

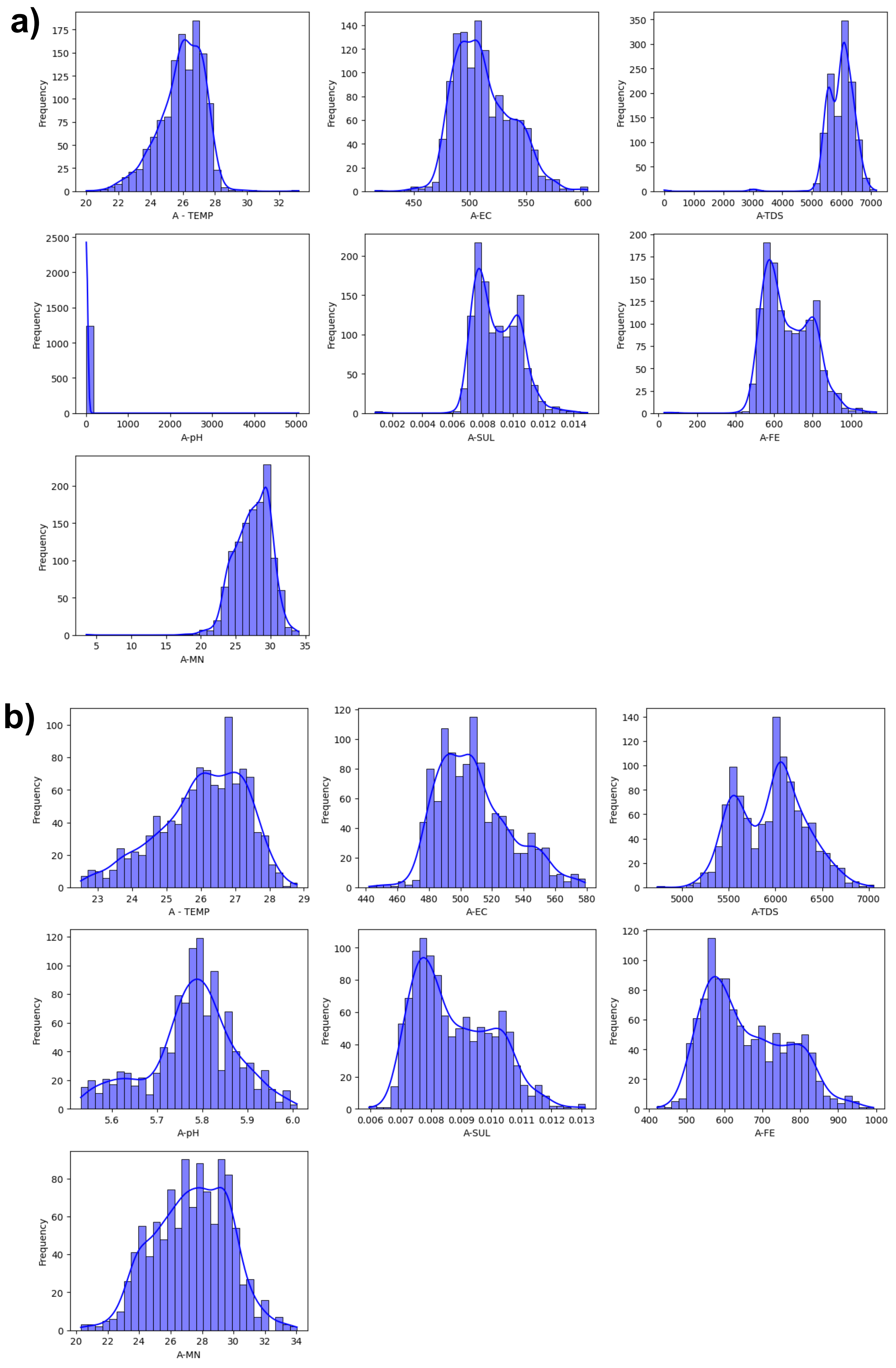

Fig. S2 *Histogram and density plot traces indicating the statistical distribution of Pump A a) Prior to outlier removal and b) After outlier removal*

* hlanganani.tutu@wits.ac.za

Table S1 : Descriptive statistics after outlier removal pertaining to Pump A.

	A - TEMP	A-EC	A-TDS	A-pH	A-SUL	A-FE	A-MN
**count**	1107.00	1107.00	1107.00	1107.00	1107.00	1107.00	1107.00
**mean**	26.03	508.64	5951.39	5.78	0.01	657.09	27.32
**std**	1.26	23.29	365.57	0.10	0.00	108.64	2.36
**min**	22.50	442.00	4730.00	5.53	0.01	421.02	20.29
**25%**	25.20	491.00	5620.00	5.73	0.01	567.35	25.59
**50%**	26.20	505.00	6002.00	5.78	0.01	631.52	27.44
**75%**	27.00	523.00	6192.00	5.83	0.01	740.93	29.19
**max**	28.80	579.00	7051.00	6.01	0.01	993.05	34.03

Table S2 : Model training and testing statistics obtained for Pump A (shaded models are the best performing models).

Model	Training		Testing		
	NMSE	Standard deviation	MSE	MAE	R^2
**LR**	-0.00605	-0.001	0.006296	0.060355	0.810643
**RD**	-0.00606	-0.001062	0.006289	0.060255	0.810865
**LASSO**	-0.03217	-0.005089	0.033252	0.153985	-6.9×10^{-5}
**EN**	-0.03217	-0.005089	0.033252	0.153985	-6.9×10^{-5}
**KNNR**	-0.00615	-0.001026	0.006372	0.061412	0.808349
**DT**	-0.01072	-0.001448	0.012552	0.089095	0.622486
**SVR**	-0.00532	-0.000763	0.005275	0.055935	0.841351
**XG**	-0.00731	-0.00123	0.007042	0.065316	0.788193
**RF**	-0.00648	-0.000935	0.006457	0.061755	0.805815
**MLP**	-0.00597	-0.001033	0.0067	0.061866	0.798483
**Stacked (All models)**	-0.00544	-0.000814	0.005252	0.055598	0.842041
**Stacked (only best models)**	-0.00546	-0.000858	0.005335	0.055679	0.839538

*hlanganani.tutu@wits.ac.za

PUMP B

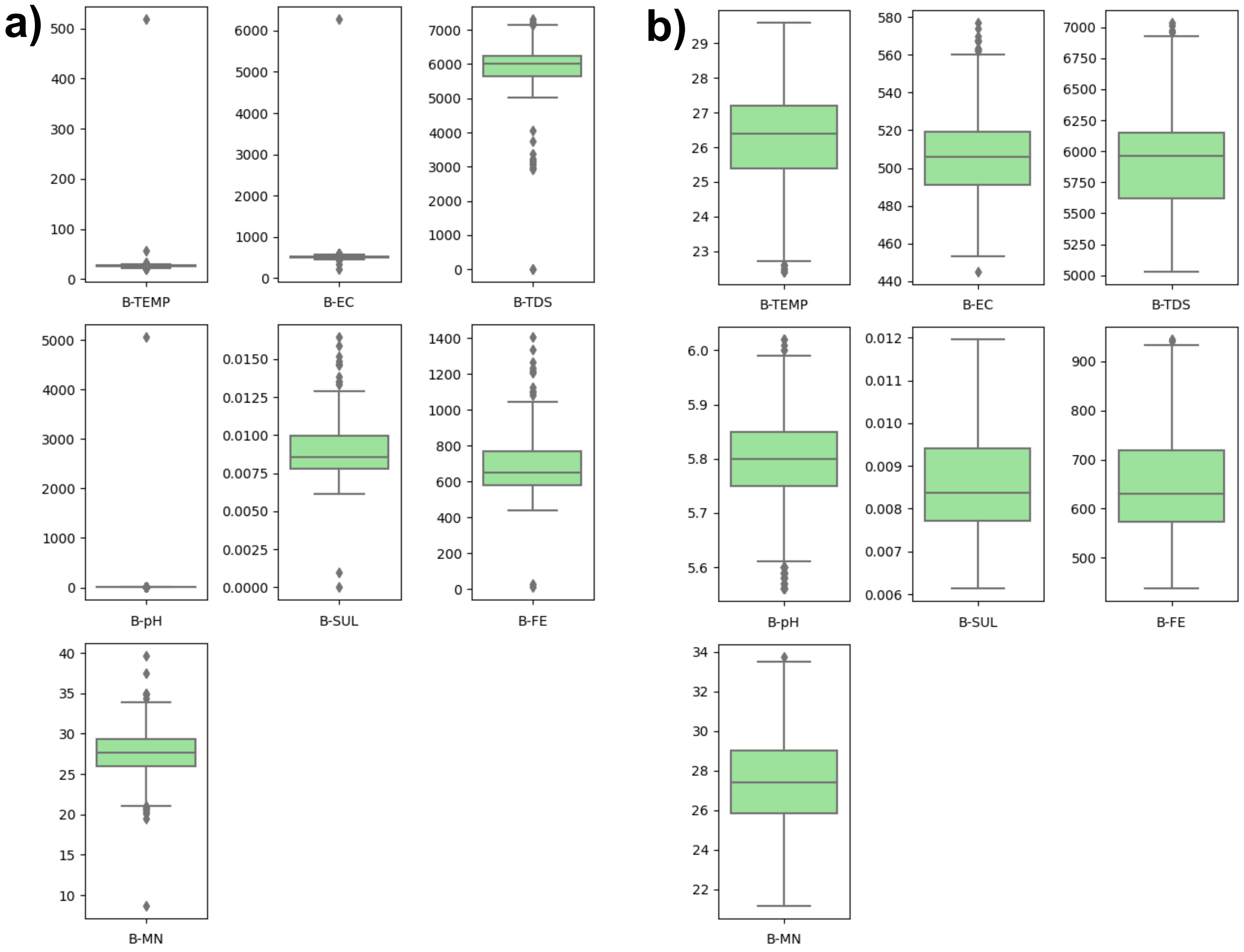

Fig. S3 *Box and whisker diagrams of Pump B indicating its statistical distribution a) Prior to outlier removal and b) After outlier removal*

*hlanganani.tutu@wits.ac.za

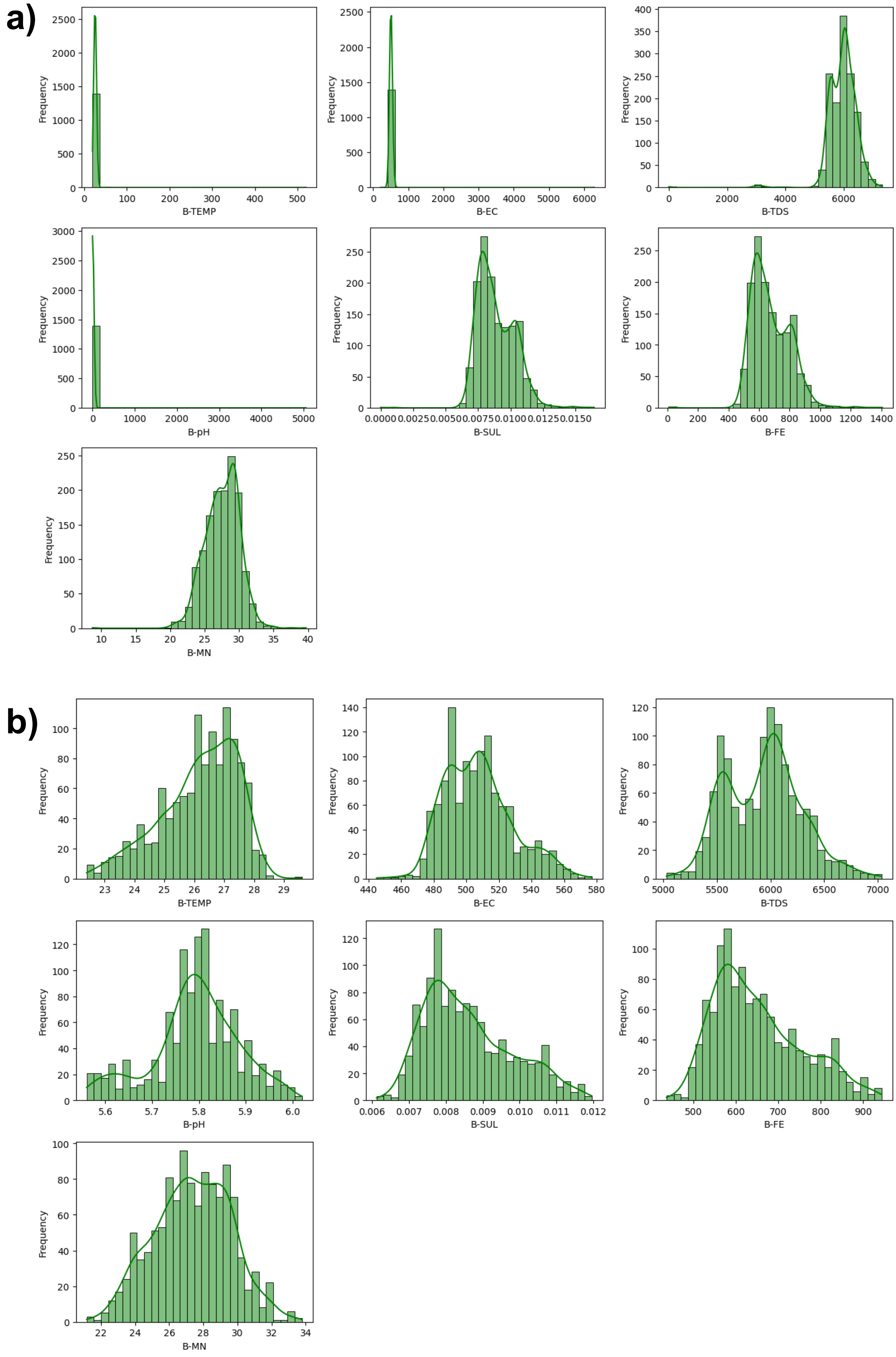

Fig. S4 *Histogram and density plot traces indicating the statistical distribution of Pump B a) Prior to outlier removal and b) After outlier removal*

*hlanganani.tutu@wits.ac.za

Table S3 : Descriptive statistics after outlier removal for Pump B.

	B-TEMP	B-EC	B-TDS	B-pH	B-SUL	B-FE	B-MN
**count**	1189.00	1189.00	1189.00	1189.00	1189.00	1189.00	1189.00
**mean**	26.16	507.48	5932.77	5.79	0.01	652.05	27.35
**std**	1.29	20.83	349.85	0.09	0.00	103.69	2.23
**min**	22.40	445.00	5028.00	5.56	0.01	436.91	21.16
**25%**	25.40	491.00	5618.00	5.75	0.01	572.65	25.84
**50%**	26.40	506.00	5965.00	5.80	0.01	630.11	27.38
**75%**	27.20	519.00	6148.00	5.85	0.01	719.43	29.01
**max**	29.60	577.00	7037.00	6.02	0.01	944.00	33.77

Table S4: Model training and testing statistics obtained for Pump B (shaded model is the best performing model).

Model	Training		Testing		
	NMSE	Standard deviation	MSE	MAE	R^2
**LR**	-1.8×10^{-5}	-0.000005	0.000013	0.002706	0.99971
**RD**	-5.7×10^{-5}	-0.000008	0.000047	0.005536	0.998901
**LASSO**	-0.04149	-0.005781	0.043197	0.169071	-0.0001
**EN**	-0.04149	-0.005781	0.043197	0.169071	-0.0001
**KNNR**	-0.00013	-0.000065	0.000086	0.006893	0.99801
**DT**	-5.1×10^{-5}	-0.000012	0.000048	0.005373	0.998883
**SVR**	-0.00242	-0.000258	0.002405	0.044632	0.944313
**XG**	-3.2×10^{-5}	-0.000009	0.000028	0.004133	0.999354
**RF**	-3.5×10^{-5}	-0.000011	0.000029	0.004299	0.999324
**MLP**	-0.00045	-0.000232	0.000487	0.016707	0.988725
**Stacked (All models)**	-1.6×10^{-5}	-0.000004	0.000011	0.002656	0.999741
**Stacked (only best models)**	-1.6×10^{-5}	-0.000005	0.000011	0.002617	0.999737

*hlanganani.tutu@wits.ac.za

TREATED WATER

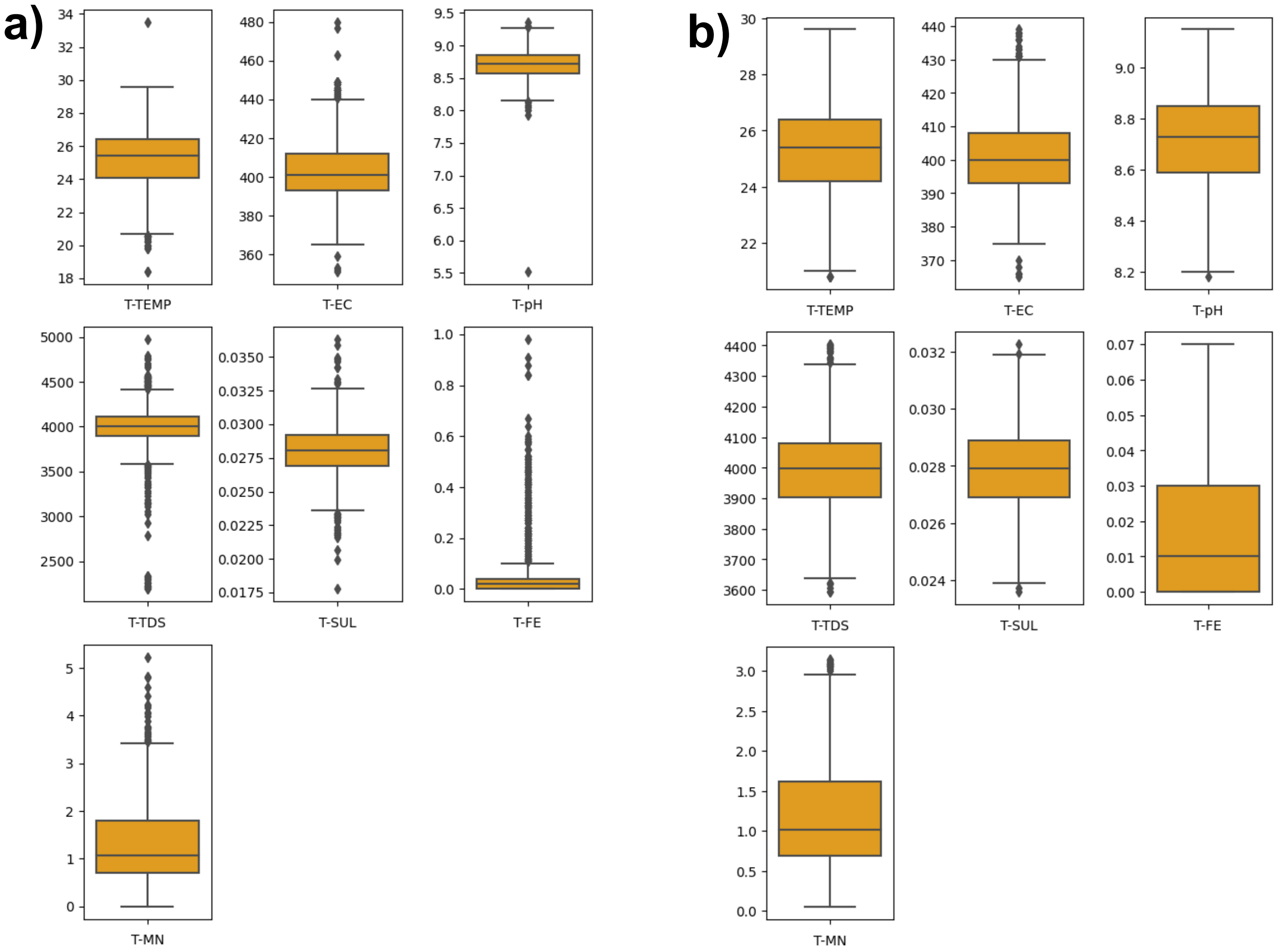

Fig. S5 *Box and whisker diagrams of the Treated Water indicating its statistical distribution a) Prior to outlier removal and b) After outlier removal*

*hlanganani.tutu@wits.ac.za

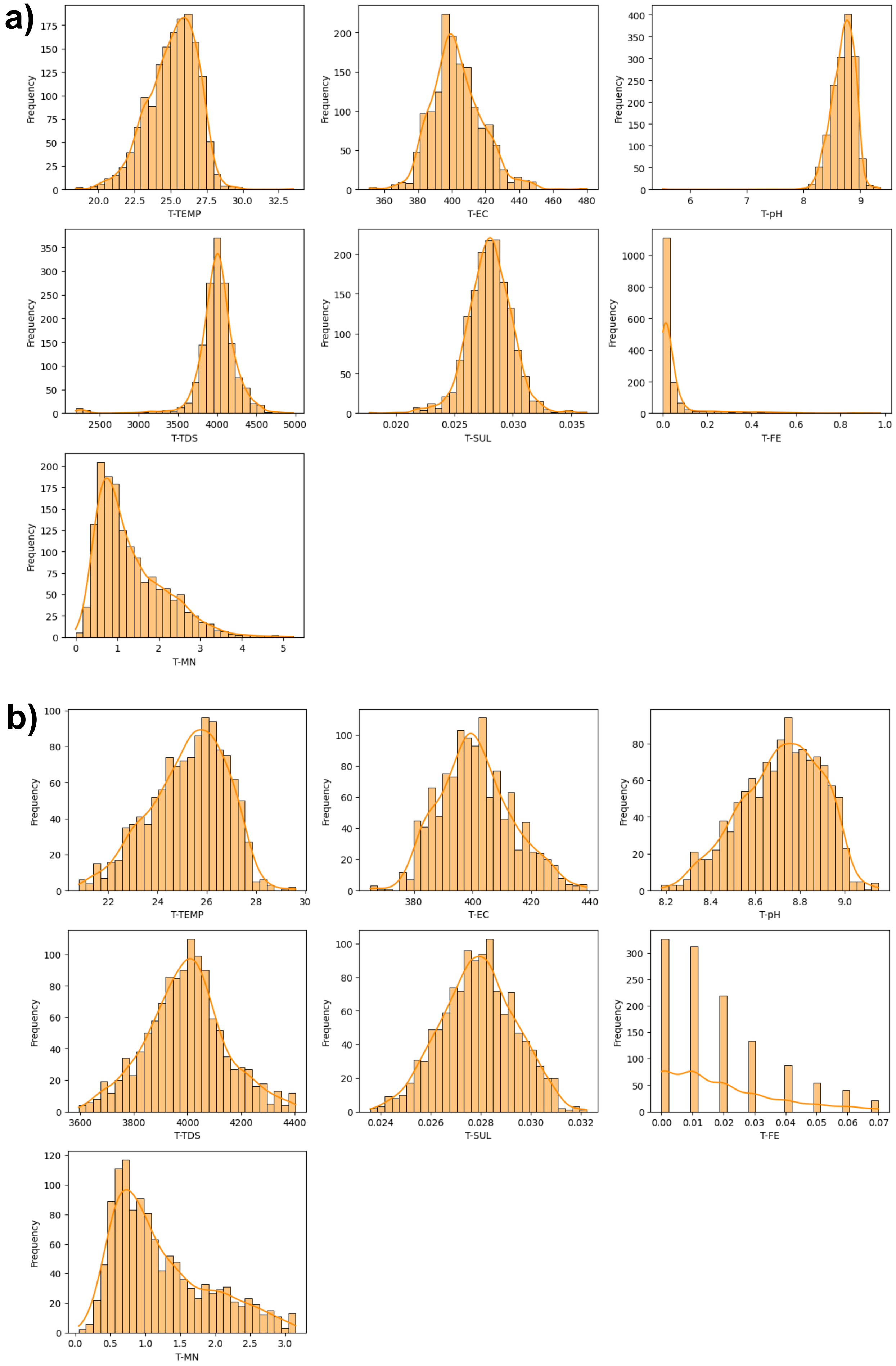

Fig. S6 *Histogram and density plot traces indicating the statistical distribution of the Treated Water a) Prior to outlier removal and b) After outlier removal*

*hlanganani.tutu@wits.ac.za

Table S5 : Descriptive statistics after outlier removal for Treated Water.

	T-TEMP	T-EC	T-pH	T-TDS	T-SUL	T-FE	T-MN
**count**	1197	1197	1197	1197	1197	1197	1197
**mean**	25.24	401.01	8.71	3994.29	0.03	0.02	1.22
**std**	1.550	12.476	0.179	148.330	0.001	0.018	0.681
**min**	20.8	365	8.18	3594	0.0236	0	0.05
**25%**	24.2	393	8.59	3902	0.02689	0	0.69
**50%**	25.4	400	8.73	3998	0.02792	0.01	1.017
**75%**	26.4	408	8.85	4079	0.0289	0.03	1.616
**max**	29.6	439	9.15	4404	0.03225	0.07	3.146

Table S6 : Model training and testing statistics obtained for Treated Water (shaded model is the best performing model).

Model	Training		Testing		
	NMSE	Standard deviation	MSE	MAE	R^2
**LR**	-0.02526	-0.003536	0.024533	0.122144	0.137285
**RD**	-0.02526	-0.003526	0.024502	0.12226	0.138378
**LASSO**	-0.02947	-0.003407	0.028459	0.134916	-0.00077
**EN**	-0.02947	-0.003407	0.028459	0.134916	-0.00077
**KNNR**	-0.02944	-0.004281	0.029781	0.133188	-0.04724
**DT**	-0.04845	-0.00349	0.055957	0.191006	-0.96773
**SVR**	-0.02518	-0.004379	0.024902	0.123406	0.124322
**XG**	-0.03296	-0.005712	0.033683	0.14398	-0.18448
**RF**	-0.02794	-0.004542	0.029278	0.133407	-0.02958
**MLP**	-0.02576	-0.003418	0.025086	0.123928	0.117861
**Stacked (All models)**	-0.02495	-0.003778	0.024362	0.121571	0.143315
**Stacked (only best models)**	-0.02501	-0.00388	0.024403	0.121403	0.141863

*hlanganani.tutu@wits.ac.za
